# Stage-Stratified Explainable Machine Learning Identifies a Reproducible Transcriptomic Classifier with Stage-Dependent Feature Importance in Colorectal Cancer: A Multi-Cohort Study with Saudi Population Contextualisation

**DOI:** 10.3390/biomedicines14102375

**Published:** 2026-10-18

**Authors:** Asif Hassan Syed, Sultan Alhayyani, Nashwan Alromema, Hatem A. Almazarqi, Altyeb Altaher Taha

**Affiliations:** 1 Department of Computer Science, Faculty of Computing and Information Technology in Rabigh, King Abdulaziz University, Jeddah 21589, Saudi Arabia; nalromema@kau.edu.sa; 2 Department of Chemistry, Faculty of Sciences and Arts in Rabigh, King Abdulaziz University, Jeddah 21589, Saudi Arabia; salhayyani@kau.edu.sa; 3 Department of Information Technology, Faculty of Computing and Information Technology in Rabigh, King Abdulaziz University, Jeddah 21911, Saudi Arabia; halmazarqi@kau.edu.sa (H.A.A.); aaataha@kau.edu.sa (A.A.T.)

**Keywords:** colorectal cancer, explainable artificial intelligence, SHapley Additive exPlanations, transcriptomics, machine learning, stage stratification, feature importance, differential expression, external validation, *ESM1*

## Abstract

**Background**: Colorectal cancer (CRC) is molecularly heterogeneous across stages, yet most transcriptomic classifiers treat it as static and rarely test whether the importance of their genes changes with disease stage. **Methods**: A stage-stratified explainable machine-learning pipeline was applied to the Cancer Genome Atlas colon adenocarcinoma cohort (286 tumour, 41 normal). A ten-gene panel was derived by cross-validated stability selection; six classifiers were benchmarked under a leakage-free protocol; and exact SHapley Additive exPlanations (SHAP) were computed within each American Joint Committee on Cancer (AJCC) stage. Findings were tested in three independent cohorts (GSE50760, GSE39582, GSE17536) and corroborated by literature, GEPIA2, and four-platform pathway enrichment. **Results**: The ten-gene panel classified tumour versus normal tissue with internal AUC up to 1.000 and external AUC 0.969 (95% CI 0.896–1.000; area under the precision-recall curve [AUPRC] 0.980), robust to class imbalance. Stage-stratified SHAP showed a monotonic shift in feature importance across stages (cross-stage Spearman rho 0.66–0.93) that replicated at three of four stages in an independent cohort (rho up to 0.94); *ESM1* rose to top-two late-stage importance in both cohorts, consistent with its role in CRC angiogenesis. The panel recovered known cancer biology, nominated novel candidates, and proved complementary to network-hub genes, and its enriched pathways matched four of five frequently altered Saudi CRC pathways. Survival discrimination was at chance (C-index 0.50–0.58), defining the panel as a classifier rather than a prognostic model. **Conclusions**: This is, to our knowledge, the first evidence in CRC that an explainable classifier’s feature-importance structure is itself stage-dependent and externally replicable. Coupling exact interpretability with reproducible, transparently scoped validation, the panel generalises as a classifier but not a prognostic model, and its convergence with Saudi CRC alterations defines conditions for prospective population-specific validation. Code and processed data are publicly available.

## 1. Introduction

### 1.1. The Clinical and Computational Burden of Colorectal Cancer

Colorectal cancer (CRC) is one of the most common malignancies worldwide and the second leading cause of cancer-related death, with GLOBOCAN 2022 estimates reporting 1,926,425 new cases and 904,019 deaths globally in a single year [1,2]. The burden is projected to rise substantially through 2050, driven largely by demographic ageing and the adoption of Westernised dietary and lifestyle patterns in transitioning economies [1,3]. Within Saudi Arabia specifically, CRC is the most prevalent cancer among men and the third most prevalent among women, with the national age-standardised incidence rate rising sharply over the past two decades and regional rates varying more than six-fold, from 3.2 per 100,000 in Jouf to 21.8 per 100,000 in Riyadh [4,5]. Approximately one-third of Saudi CRC patients are diagnosed at an advanced stage [6], with some registry-based estimates placing the proportion of late-stage presentation with regional or distal metastasis as high as 50% [7]. National colonoscopy screening uptake for the recommended 45–75-year age band remains below 10%, and fewer than 1% of older adults undergo the procedure annually [5]. This combination of rising incidence, late-stage presentation, and low screening uptake creates a measurable and quantifiable gap between the disease burden and the diagnostic and prognostic tools currently available to Saudi clinicians—a gap that is computational and translational in nature, not merely a question of healthcare access.

Histopathological staging (TNM) remains the dominant prognostic framework in routine CRC practice, yet it captures anatomical extent of disease without resolving the underlying molecular heterogeneity that drives markedly different clinical trajectories among patients sharing the same stage [8]. Molecular subtyping efforts, including consensus molecular subtypes (CMS) derived from gene expression, have demonstrated that transcriptomic state carries prognostic information beyond TNM stage alone [9], motivating a substantial and ongoing research effort to identify transcriptomic biomarkers capable of refining diagnosis, prognosis, and treatment stratification in CRC. Large public repositories—principally The Cancer Genome Atlas (TCGA-COAD) and the National Center for Biotechnology Information Gene Expression Omnibus (NCBI GEO)—have made tens of thousands of CRC transcriptomic profiles freely available, creating the data substrate for this effort but also exposing a persistent methodological problem: differential expression analysis alone identifies long, often unstable, gene lists with limited mechanistic interpretability and inconsistent reproducibility across cohorts [10,11]. TCGA-COAD itself includes AJCC pathologic stage annotation for most of its cohort, with documented per-stage sample counts (Stage I, *n* = 44; Stage II, *n* = 110; Stage III, *n* = 83; Stage IV, *n* = 40 in representative curated subsets), providing sufficient per-stage depth for stage-resolved computational analysis.

### 1.2. The Rise of Explainable AI in Transcriptomic Biomarker Discovery, and Its Unresolved Limitations

Machine learning models, and gradient-boosted tree ensembles such as XGBoost in particular, have become the dominant approach for transcriptomic biomarker classification because of their capacity to model non-linear, high-order interactions among thousands of candidate genes while resisting overfitting through regularisation [11,12]. A recent representative study combining differential expression analysis with two-sample Mendelian randomisation and nine machine learning algorithms on TCGA and GEO (GSE87211) CRC data reported that XGBoost achieved an AUC of 0.990 in distinguishing CRC from normal tissue, with SHAP (SHapley Additive exPlanations) analysis subsequently identifying *IFITM1* and *DBNDD1* as the most influential genes driving model predictions [13]. A separate machine learning study integrating four feature-selection algorithms with four classifiers on TCGA and GEO RNA-seq data similarly used SHAP-derived co-predictive networks, visualised via VisuNet and cross-referenced against the STRING protein–protein interaction (PPI) database, to interpret which genes most strongly informed CRC tumour micro-environment classification [14]. Beyond tissue transcriptomics, SHAP has also been applied to circulating proteomic data: a 2025 UK Biobank study using LASSO, XGBoost, and LightGBM classifiers identified *TFF3*, *LCN2*, and *CEACAM5* as SHAP-prioritised plasma biomarkers linked to cell adhesion and inflammatory signalling pathways [15]. Collectively, these studies establish SHAP-based explainability as the prevailing standard for rendering gradient-boosted CRC classifiers interpretable at the global, cohort-average level.

However, a global SHAP ranking computed across an entire cohort, irrespective of tumour stage, implicitly assumes that the biological drivers of malignancy remain constant across the natural history of the disease—an assumption that recent stage-resolved explainability work directly contradicts for clinical and demographic features. A 2026 multi-cancer survival study applying SHAP and LIME to CatBoost models trained separately on localised, regional, and distant-stage colorectal, stomach, and liver cancer cohorts found that the top-ranked predictors shift substantially by stage: tumour size and surgical code dominate at the localised stage, nodal indicators and radiation become prominent at the regional stage, and histologic subtype codes emerge as dominant only at the distant-metastatic stage [16]. This finding, while compelling, was derived entirely from registry-level clinical and demographic covariates (age, tumour extension, nodal counts, and treatment codes) rather than from molecular transcriptomic features, leaving open the question of whether an analogous stage-dependent reordering of importance occurs at the gene-expression level. Independently, a 2025 whole-transcriptome study of formalin-fixed paraffin-embedded tissue across the adenoma, carcinoma in situ, and adenocarcinoma progression sequence confirmed, via gene-set enrichment and RT-qPCR validation, that distinct transcriptomic profiles characterise each stage of CRC progression [17], and earlier stage-specific network and differential-expression studies identified distinct sets of driver genes associated with progression to stage II, III, and IV disease using centrality and co-expression methods [18], with real-time PCR validation confirming that genes such as FOXM1, hTERT, and MCTP1 display stage-dependent expression patterns across the adenoma-to-carcinoma sequence [19]. None of these stage-specific transcriptomic studies, however, applied a SHAP-based explainable-AI framework that quantifies, within a single trained predictive model, how gene-level importance redistributes across Stage I through Stage IV—the precise analytical step that would bridge the clinical-covariate stage-explainability literature [16] with the stage-specific transcriptomic literature [17,18,19].

### 1.3. Multi-Omics Integration and the Saudi Context: What Is Available, and What Is Not

The broader push toward multi-omics integration in CRC—combining transcriptomic, proteomic, metagenomic, and radiomic layers—has been proposed as the route toward a more complete, dynamic understanding of tumour heterogeneity that single-omics, gene-centric models cannot capture [20]. In practice, however, the great majority of published CRC transcriptomic biomarker and SHAP-explainability studies draw exclusively on TCGA and Western or East Asian GEO cohorts [13,14,15], a pattern consistent with the broader under-representation of Middle Eastern and North African populations in large-scale cancer genomics research. This is a substantive limitation rather than a cosmetic one: Saudi CRC epidemiology differs materially from the Western populations underlying most public reference cohorts, with a markedly younger median age at diagnosis, a high proportion of early-onset disease, and the late-stage presentation pattern noted above [4,5,6,7]. A biomarker panel and an associated explainability profile derived without any consideration of a population with this distinct epidemiological signature leaves an open question about contextual relevance that a purely TCGA/GEO-trained study cannot itself answer.

It is important to state plainly what is, and is not, publicly available to address this question. No large-scale, freely downloadable transcriptomic (gene-expression) dataset from a confirmed Saudi-national CRC patient cohort currently exists in NCBI GEO, ArrayExpress, or comparable repositories; the present authors verified this directly rather than assuming it, after an earlier accession number considered for this purpose (GSE22598) was confirmed on inspection to derive from a Japanese cohort study of UNC5B expression in colorectal cancer, unrelated to the Saudi population. What does exist publicly is a dedicated, peer-reviewed targeted next-generation sequencing study of 107 Saudi Arabian CRC patients without a family history of disease, recruited from King Fahad Medical City and King Abdullah University Hospital, reporting actionable mutation frequencies including *BRCA2* (79%), *CHEK1* (78%), *ATM* (76%), *PMS2* (76%), *ATR* (74%), and *MYCL* (73%) [21]. This is mutational (DNA-level) data, not transcriptomic (RNA expression) data, and it cannot be merged into a transcriptomic training pipeline; treating it as interchangeable with expression data would itself be a methodological error. It can, however, serve a different and legitimate purpose: as an independent, population-specific point of biological comparison against which the genes prioritised by the present study’s SHAP-based transcriptomic model can be checked for pathway-level convergence, without any claim that the model was trained on, or validated against, Saudi expression data. This distinction is maintained explicitly throughout the present study.

A further limitation common to the SHAP-explainability literature in CRC concerns survival modelling. Most studies in this space report classification metrics (AUC, accuracy) for a binary tumour-versus-normal or stage-versus-stage task [13,14,15], but stop short of benchmarking modern survival-specific architectures—such as DeepSurv or Random Survival Forests—against the conventional Cox proportional hazards model using the same SHAP-prioritised gene panel, nor do they externally validate the resulting risk score on a fully independent cohort using concordance index and time-dependent AUC with formal statistical comparison (DeLong’s test) against a baseline. Reporting standards bodies, including the TRIPOD-AI initiative for clinical prediction models, increasingly regard bootstrapped confidence intervals, external cohort validation, and explicit calibration assessment as minimum requirements for a clinically credible AI-based prognostic tool [22], yet these elements remain inconsistently applied across the CRC transcriptomic SHAP literature reviewed above.

### 1.4. Gaps in the Existing Evidence Base

Table 1 functions not as a conventional literature summary but as a structured checklist of methodological gaps in the field. Each of the studies included here was chosen because it bears directly on one of the methodological decisions made in this paper, whether that is how features were selected, how the model was interpreted, which biological pathways were implicated, or how results were validated. For each study, the table records strengths, methodological shortcomings, and potential sources of bias; the final column, the most directly relevant to this work, identifies the specific question left open by each study that the present stage-stratified XAI pipeline was designed to address. A narrative summary follows for readers preferring a condensed overview; the table provides the complete paper-by-paper reasoning.

Collectively, the literature review reveals four convergent conclusions. First, SHAP-based explainability has been applied successfully to CRC transcriptomic and proteomic classifiers, but always as a single, cohort-averaged ranking rather than one resolved by tumour stage [13,14,15]. Second, stage-dependent shifts in predictive feature importance have been demonstrated for clinical and demographic covariates in CRC survival models, and stage-dependent transcriptional patterns have been confirmed independently at the gene-set and individual-gene level by gene-set enrichment, qRT-PCR, and network analysis—but none of these studies has been joined with SHAP-based explainable modelling [16,17,18,19]. Third, no publicly available transcriptomic dataset exists from a confirmed Saudi CRC patient cohort; the dedicated Saudi CRC molecular resource that does exist publicly is a targeted mutation (NGS) panel, not an expression dataset, and the present study uses it only as an independent point of pathway-level comparison rather than as training data [21]. Fourth, survival-model benchmarking and external validation following current reporting standards remain inconsistently applied across this literature [22], leaving open the comparison between conventional Cox proportional hazards modelling and modern survival-specific architectures on an SHAP-prioritised gene panel.

### 1.5. Research Gaps and Contributions of This Study

The foregoing review identifies four specific, interrelated gaps that motivate the present study. Each is stated explicitly below, paired with the corresponding contribution through which this study addresses it.

Gap 1—Global, cohort-averaged SHAP attribution conceals stage-dependent shifts in transcriptomic feature importance: Existing SHAP-based CRC transcriptomic classifiers report a single, stage-agnostic importance ranking [13,14,15], while stage-resolved explainability evidence to date is confined to clinical and demographic covariates rather than gene expression [16], and stage-specific transcriptomic studies that do exist use gene-set enrichment or network-centrality methods rather than SHAP-based feature attribution [17,18]. Whether, and how, SHAP-derived gene-level attribution reorders across Stage I–IV CRC within a single transcriptomic model has not been reported. Addressed by Contribution 1.

Gap 2—SHAP-based CRC transcriptomic biomarker studies are conducted without any reference to Saudi population-specific molecular data, and no public Saudi transcriptomic cohort exists to close this gap directly: Saudi CRC epidemiology differs materially from the Western cohorts underlying TCGA and most GEO datasets [4,5,6,7], yet no published SHAP-based transcriptomic biomarker study situates its findings against any Saudi-specific molecular evidence, even at the level of pathway comparison. A dedicated Saudi CRC mutation panel exists publicly [21], but it has not previously been used as a contextual comparison point for any SHAP-prioritised transcriptomic gene set. Addressed by Contribution 2.

Gap 3—Mechanistic triangulation of SHAP-prioritised genes against protein–protein interaction and pathway evidence is incomplete: Prior work has linked SHAP-prioritised genes to PPI networks or to a single enrichment database in isolation [13,14], but a triangulated approach combining high-confidence STRINGdb network topology with cross-referenced enrichment across DAVID, KEGG, Reactome, and ClueGO for the same SHAP-prioritised, stage-stratified gene set has not been reported for CRC. Addressed by Contribution 3.

Gap 4—Survival-model benchmarking and externally validated, statistically rigorous performance reporting are inconsistently applied: Most SHAP-based CRC transcriptomic studies report classification AUC on an internal split rather than benchmarking survival-specific architectures (DeepSurv, Random Survival Forest) against Cox proportional hazards on the SHAP-prioritised panel, with external cohort validation, DeLong testing, and bootstrapped confidence intervals as recommended under TRIPOD-AI [22]. Addressed by Contribution 4.

To address these four gaps, this study makes the following contributions:

Contribution 1—Stage-stratified SHAP attribution framework: The first application of SHAP attribution computed separately within each AJCC stage subgroup (I–IV) of a single CRC transcriptomic classifier—the empirically selected model from a six-algorithm benchmark, an interpretable Elastic Net—quantifying how gene-level importance redistributes across disease progression, testing concordance against global, stage-agnostic SHAP rankings, and confirming that the stage-dependent importance structure replicates in an independent cohort (addresses Gap 1).

Contribution 2—Saudi population contextualisation via independent mutational cross-reference: Following SHAP-based prioritisation of stage-stratified genes from TCGA-COAD and international GEO cohorts, the resulting enriched-pathway set is cross-referenced in the Discussion against the documented Saudi colorectal-cancer molecular landscape, including both a targeted mutation panel [21] and population-level whole-exome pathway frequencies, assessing pathway-level convergence (for example, at the Wnt/β-catenin and PI3K axes) between the transcriptomically enriched pathways and independently reported Saudi alterations, and reporting non-convergences transparently, without overstating this as transcriptomic validation in a Saudi cohort (addresses Gap 2).

Contribution 3—Triangulated mechanistic validation: A two-level mechanistic analysis in which a high-confidence (STRINGdb interaction score > 0.9) protein–protein interaction network and triangulated enrichment across DAVID, KEGG, Reactome, and ClueGO are established on the full differentially expressed gene set, and the stage-stratified SHAP-prioritised panel is then located within that well-powered network and pathway landscape. This provides convergent rather than single-database mechanistic evidence and reveals that the explainable-AI signature is complementary to, rather than a rediscovery of, classical network-hub genes (addresses Gap 3).

Contribution 4—Survival-model benchmarking with externally validated statistical rigour: Benchmarking of DeepSurv and Random Survival Forest against Cox proportional hazards on the SHAP-prioritised panel, with performance reported as concordance index and time-dependent AUC and externally validated on independent, fully open-access GEO cohorts (GSE39582, GSE17536) using bootstrapped (1000-iteration) 95% confidence intervals, in line with TRIPOD + AI reporting standards. This benchmarking is coupled to a pre-registered events-per-variable assessment that transparently delineates the signature’s scope, distinguishing a reproducible classifier from a prognostic model rather than overstating prognostic utility (addresses Gap 4).

The remainder of this paper is organised as follows. Section 2 details the cohorts, the differential-expression and network analyses, the six-algorithm classification benchmark, the stage-stratified explainability and survival modelling, and the reproducibility framework. Section 3 reports the results of each phase, Section 4 discusses their interpretation, novelty, limitations and future directions, and Section 5 concludes.

## 2. Materials and Methods

**Figure 1:**
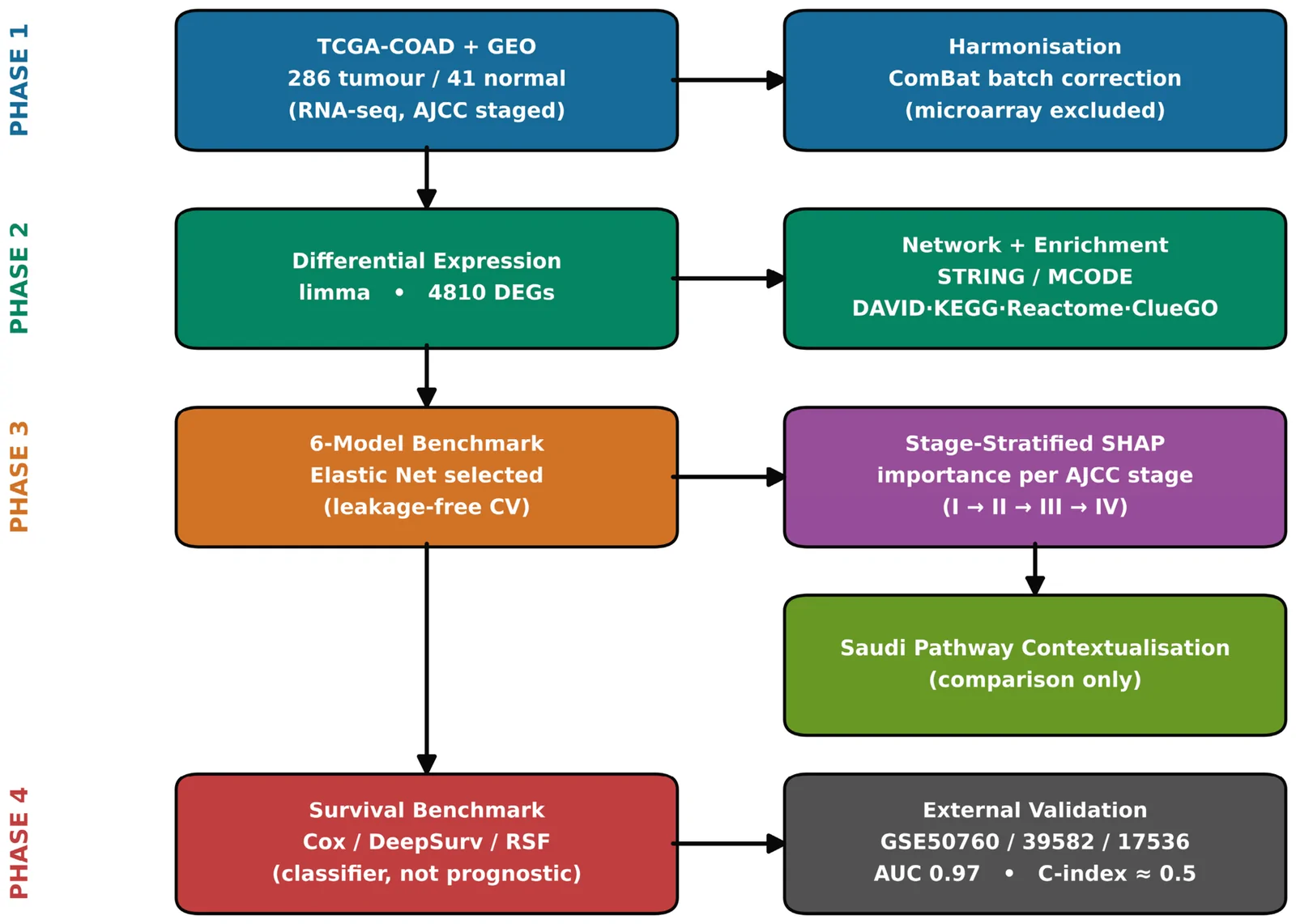
Schematic overview of the stage-stratified explainable AI (XAI) pipeline for colorectal cancer (CRC) transcriptomic biomarker discovery. Phase 1: RNA-seq-only multi-cohort data acquisition (TCGA-COAD primary tumours with both expression and stage annotation, *n* = 286, with confirmed AJCC stage distribution Stage I = 45, II = 110, III = 80, IV = 39 plus 12 unstaged, alongside 41 adjacent solid-tissue normal samples; pooled RNA-seq GEO cohorts) and harmonisation via ComBat batch correction [24], with platform-mixing risk [25] explicitly avoided by excluding microarray cohorts from the training matrix. Phase 2: differential expression analysis (limma [26,27]) combined with graph-based protein–protein interaction (PPI) network analysis (STRINGdb [28], confidence > 0.9; MCODE [29] hub detection) and triangulated enrichment across DAVID [30], KEGG [31], Reactome [32], and ClueGO [33]. Phase 3, the core novel contribution: a six-algorithm benchmark (Elastic Net [34], Random Forest [35], SVM [36], XGBoost [12], LightGBM [37], shallow MLP [38]) under identical cross-validation folds, with the empirically best-performing model (the Elastic Net, selected as the most parsimonious of a statistically tied set) carried forward for SHAP [23] attribution computed separately within each AJCC stage subgroup, yielding stage-resolved importance rankings tested for concordance against the global, stage-agnostic ranking. An independent Saudi population contextualisation step, in which the SHAP-prioritised gene set is compared at the pathway level against published Saudi-specific CRC mutation frequencies [21], explicitly not used as training or validation data. Phase 4: survival-model benchmarking (Cox proportional hazards vs. DeepSurv [39] vs. Random Survival Forest [40]) on the SHAP-prioritised panel, with external validation on the freely available GSE39582/GSE17536 cohorts [41] (replacing ICGC following the closure of open ICGC data access [42]) using concordance index [43], time-dependent AUC, DeLong’s test [44], and 1000-iteration bootstrapped 95% confidence intervals. All code, trained models, and Colab notebooks reproducing every step are released on GitHub under an MIT licence (https://github.com/SAH-ML/crc-stage-stratified-xai, accessed on 11 October 2026).

Figure 1 provides a schematic overview of the complete four-phase pipeline. Starting from harmonised, platform-consistent transcriptomic data, the pipeline applies differential expression and graph-based pathway discovery, a benchmarked comparison of six classification algorithms, stage-stratified explainable AI modelling on the empirically best-performing model, an independent Saudi population contextualisation step, and survival benchmarking with external validation, producing a fully reproducible set of outputs including stage-resolved SHAP attribution maps and an externally validated risk score. Each phase is described in full methodological detail in the subsections below, with explicit justification of every modelling choice—machine learning, deep learning, or graph-based—at the point where it is introduced, and with explicit resolutions to eight methodological risks identified prior to data acquisition (Section 2.9).

### 2.1. Rationale for Method Selection: Machine Learning, Deep Learning, and Graph-Based Analysis

Because this pipeline combines several distinct method families, the rationale for each is stated explicitly here and summarised in Table 2, rather than left implicit, so that the choice of tool is transparent and auditable at every stage rather than assumed. Critically, the primary classifier is not assumed in advance: Section 2.5.1 specifies a six-algorithm benchmark, and the model carried forward for SHAP attribution is the empirical winner of that benchmark, not a default choice.

### 2.2. Study Cohorts and Data Acquisition

#### 2.2.1. Training and Internal Validation Cohorts

Primary training data were obtained from the Cancer Genome Atlas Colon Adenocarcinoma project (TCGA-COAD), comprising RNA-sequencing expression profiles and matched clinical annotation, including AJCC pathologic stage [8]. The complete TCGA-COAD clinical matrix contained 551 samples and 133 clinical variables, and the expression matrix provided 20,530 genes. After restricting to primary tumour samples carrying both a usable expression profile and a documented AJCC stage, 286 tumour samples were retained, with the confirmed stage distribution Stage I (*n* = 45), Stage II (*n* = 110), Stage III (*n* = 80), and Stage IV (*n* = 39), together with 12 tumours of indeterminate stage that are excluded from stage-stratified analyses; 41 adjacent solid-tissue normal samples with expression data serve as the tumour-versus-normal comparison group. Sub-stages were collapsed to their parent AJCC stage (for example, Stage IIA, IIB, and IIC to Stage II; Stage IIIA, IIIB, and IIIC to Stage III) prior to all stage-stratified analyses, and three samples annotated “[Discrepancy]” were treated as unstaged. These counts are the empirically confirmed values obtained during data acquisition for this study and supersede any preliminary planning estimates.

Data were retrieved directly from the UCSC Xena platform [45], which mirrors TCGA data without requiring an institutional data use agreement. Three files were downloaded from the Xena TCGA hub (https://xenabrowser.net/datapages/?dataset=TCGA.COAD.sampleMap/HiSeqV2&host=https://tcga.xenahubs.net, accessed on 11 July 2026): the gene-level RNA-seq expression matrix (dataset TCGA.COAD.sampleMap/HiSeqV2; values are log2(norm_count+1)), the clinical/phenotype matrix (TCGA.COAD.sampleMap/COAD_clinicalMatrix), and the curated survival endpoint file (survival/COAD_survival.txt, providing overall survival status and time used in Phase 4). The exact download URLs, file checksums, retrieval date, and the precise sample-filtering steps that yield the counts above are recorded in the accompanying GitHub repository so that the cohort can be reconstructed identically by any reader. Following the cross-platform integration risk identified during methodological review (Section 2.9, Issue 1), training data acquisition is restricted to RNA-seq cohorts only; candidate GEO cohorts were screened to exclude microarray-based series from the training matrix, retaining microarray cohorts solely for the separate, explicitly labelled external validation role described in Section 2.7.2. The AJCC staging edition associated with each cohort’s clinical metadata [8] is recorded at this stage, and any cohort lacking a documented edition is conservatively remapped to AJCC 8th-edition stage groupings before stage-stratified analysis (Section 2.9, Issue 7).

The TCGA-COAD survival file provided overall-survival annotation for 545 patients with 123 recorded deaths (events) across the full cohort. The number of events within each AJCC stage subgroup is tabulated and reported in the Results prior to any stage-specific survival modelling, so that compliance with the events-per-variable heuristic [46] can be assessed transparently rather than assumed (Section 2.9, Issue 3).

#### 2.2.2. Two-Tier External Validation Cohorts

To address the two distinct validation questions that arise for a model trained on a single platform—first, whether the discovered signature replicates on independent data of the same platform, and second, whether it generalises across platforms—external validation is performed at two tiers using independent cohorts, none of which is used at any point during feature selection, model training, or hyperparameter tuning. This separation is enforced at the data-loading level in the analysis code: validation cohorts are not loaded into memory until the final model, feature panel, and SHAP attributions are locked.

Tier 1 (same-platform replication): GSE50760 [47], an independent Illumina RNA-seq CRC cohort comprising 18 primary tumour and 18 matched normal-colon samples, is used to test whether the tumour-versus-normal classification signature replicates on RNA-seq data generated by a different laboratory. Because this cohort and the TCGA-COAD training cohort share the same measurement platform (RNA-seq), this tier isolates biological replication from any platform-transfer confound. It is stated transparently that GSE50760 lacks AJCC stage annotation, and its tumours derive from patients with metastatic disease; it therefore validates the tumour-versus-normal classification signature only and does not validate the stage-stratified attribution or survival components, which are addressed at Tier 2.

Tier 2 (cross-platform generalisation, stage-stratified, and survival validation): GSE39582 and the GSE17536/GSE17537 series [41], large, fully open-access microarray CRC cohorts with complete clinical, TNM stage, and survival annotation that have been used as external validation cohorts in numerous published CRC prognostic studies. GSE39582 in particular provides full TNM staging for a large cohort (reported distributions include stage I/II, stage III with N > 200, and stage IV), which makes it suitable not only for overall signature generalisation and survival validation but also—importantly—for external validation of the stage-stratified attribution claim that is the central novelty of this study. Accordingly, the stage-stratified SHAP-prioritised gene panel is transferred to GSE39582 (via the cross-platform signature-transfer protocol of Section 2.2.3), and its discriminative behaviour is evaluated within each available TNM stage subgroup, providing an external, independent test of whether the stage-dependent importance pattern observed internally on TCGA-COAD reproduces in a separate cohort. This tier therefore tests three distinct questions: (i) does the signature generalise beyond the discovery platform; (ii) does the stage-dependent attribution pattern replicate externally; and (iii) does the SHAP-prioritised panel carry the survival signal evaluated in Phase 4. The combination of a same-platform RNA-seq cohort (Tier 1) with cross-platform, fully-staged microarray cohorts (Tier 2) constitutes a layered validation design in which each tier answers explicitly stated questions; a recent peer-reviewed CRC biomarker study adopted precisely this TCGA-RNA-seq-plus-GSE39582/GSE17536-microarray configuration, establishing direct precedent for the cross-platform component [41].

It is stated transparently that this external stage-stratified validation is cross-platform (microarray validation of an RNA-seq-derived signature) rather than same-platform: no public RNA-seq CRC cohort combining adequate per-stage sample sizes with complete four-stage annotation was identified at the time of writing, so same-platform external stage validation was not achievable with existing public data. This residual constraint is inherent to current data availability rather than to study design, and the cross-platform stage validation on GSE39582 represents the strongest external stage-level test the available public data permit.

These cohorts replace the originally planned International Cancer Genome Consortium (ICGC) COAD-READ cohort, following confirmation that the ICGC Data Portal was retired in 2024 and that ICGC controlled-access data—including the detailed clinical and survival annotation required for this study—now requires Data Access Compliance Office (DACO) approval rather than being freely downloadable [42].

#### 2.2.3. Cross-Platform Signature Transfer Protocol

Because RNA-seq (TCGA-COAD, GSE50760) and microarray (GSE39582, GSE17536/GSE17537) quantify expression on different numerical scales with different dynamic ranges and noise structures, the trained model’s raw decision thresholds are not applied directly to microarray values, as doing so would compare incomparable scales and would constitute a genuine methodological error. Instead, cross-platform validation operates at the level of the gene signature, not the raw model: the SHAP-prioritised gene panel identified on TCGA-COAD is transferred to each microarray cohort, where all expression values are first z-score standardised per gene within each cohort to place platforms on a comparable, mean-centred footing. For external validation of the stage-stratified attribution specifically, the primary model is a linear (Elastic Net) classifier, for which the SHAP contribution of each gene is exactly the product of the model’s learned coefficient and the gene’s mean-centred (z-scored) value. The transferred per-gene attribution in the target cohort is therefore computed exactly as this coefficient-times-value product, and stage importance is the mean absolute attribution within each TNM stage subgroup, mirroring the discovery-cohort computation precisely. This exact linear-attribution transfer is used in preference to recalibrating a separate classifier on pseudo-labels in the target cohort; a diagnostic comparison (released in the code) confirmed that the exact attribution and a variance-weighted check agree closely (Spearman ρ ≈ 0.97), whereas a pseudo-label recalibration introduced avoidable distortion and understated cross-cohort agreement. This tests the scientifically meaningful question of whether the same genes retain their stage-resolved importance structure on an independent platform, rather than the ill posed question of whether identical numerical thresholds transfer across incompatible scales. For the same-platform Tier 1 cohort (GSE50760), where scales are directly comparable after standard normalisation, the trained model can additionally be applied with only per-gene standardisation, and both results are reported. Per-gene z-score standardisation places each gene on a common, platform-independent relative scale (standardised within each cohort), and the linear-additive structure of the Elastic Net makes the resulting attribution mathematically well-defined under this transformation; this is why the gene signature, rather than the raw model, is transferred across platforms. We note, however, that this strategy mitigates but does not fully resolve probe-to-gene mapping ambiguity and differences in dynamic range between platforms, which remain a residual cross-platform limitation (Section 4.6). The importance of such modality-specific considerations when transferring predictive models into clinical staging contexts has been emphasised in recent work [48,49].

#### 2.2.4. Saudi Population Comparison Data

A previously published, peer-reviewed targeted next-generation sequencing study of 107 Saudi Arabian CRC patients without family history of disease, recruited from King Fahad Medical City and King Abdullah University Hospital, reported actionable mutation frequencies for a curated cancer gene panel [21]. This study is used exclusively as a literature-derived comparison point in Section 2.6 and Section 4; no individual-level data, raw sequencing files, or expression values from this cohort enter the present analysis pipeline at any stage. No transcriptomic dataset from a confirmed Saudi-national CRC patient cohort was identified in any public repository at the time of writing; this absence is stated explicitly rather than worked around.

### 2.3. Data Preprocessing and Quality Control

#### 2.3.1. Probe and Sample Filtering

Genes with zero or near-zero variance across samples and genes with missing expression values in more than 10% of samples in any single cohort were removed prior to integration. Samples with fewer than 70% of genes successfully measured, or flagged as low-quality by the originating study’s own quality-control pipeline (where reported), were excluded.

#### 2.3.2. Cross-Cohort Batch Correction

Because TCGA-COAD and the supplementary RNA-seq GEO cohorts were generated at different times and by different laboratories, merging them directly would confound biological signal with technical batch effects. ComBat empirical Bayes batch correction [24] (implemented via the pycombat Python package) was applied using cohort-of-origin as the batch covariate, with disease status (tumour/normal) and AJCC stage specified as variables to be protected from over-correction. Batch correction was validated visually using principal component analysis (PCA) and t-distributed stochastic neighbour embedding (t-SNE) projections generated before and after correction; successful correction was defined as the disappearance of cohort-of-origin clustering while tumour-versus-normal and stage-related clustering remained visually distinguishable. Restricting this step to RNA-seq-only cohorts directly addresses the documented risk that mixing microarray and RNA-seq platforms can reduce the common, batch-correctable feature space to fewer than 100 genes [25], which would have constrained both model capacity and biological interpretability had it been allowed to occur unaddressed.

#### 2.3.3. Stage-Stratified Data Partitioning

Following preprocessing, the harmonised training cohort was partitioned for nested cross-validation using stratified sampling that preserved both disease status and AJCC stage proportions within each fold, ensuring that no stage subgroup was systematically over- or under-represented in any training or inner-validation partition. The 12 tumours that were not staged (“[Discrepancy]”) were only not included in the stage-stratified SHAP analysis, which requires a staging label, and included in all other analyses that do not require staging. The tumour-versus-normal classifier (without the stage label) is applicable to unstaged cases, which are common in routine clinical data, whereas the stage-stratified attribution is only valid for staged cases.

#### 2.3.4. Prevention of Information Leakage

A recognised and frequently overlooked source of optimistic bias in transcriptomic machine-learning studies is information leakage, in which steps that should be confined to the training data are instead performed on the entire dataset before cross-validation, allowing the held-out folds to influence model development indirectly. To prevent this, every data-dependent transformation in this study is fitted inside the cross-validation loop, on each outer fold’s training partition only, and then applied to the corresponding held-out fold without refitting. This applies specifically to: (i) differential-expression-based and any other feature selection, which is recomputed from scratch within each training fold so that the held-out fold never participates in choosing features; (ii) batch-effect correction parameters; and (iii) any per-gene normalisation or standardisation statistics. The single trained model and its locked feature panel are only then taken to the fully independent external cohorts (Section 2.2.2), which are never loaded during training, tuning, or feature selection. This nested arrangement ensures that all reported internal-validation performance estimates are free of the feature-selection and preprocessing leakage that would otherwise inflate them and is implemented and verifiable in the released analysis code.

### 2.4. Differential Expression Analysis and Graph-Based Mechanistic Discovery

#### 2.4.1. Differential Expression Analysis

Differentially expressed genes (DEGs) between tumour and adjacent-normal samples were identified using the limma framework (https://bioconductor.org/packages/limma accessed on 11 July 2026) [26,27]. Because the TCGA-COAD expression matrix obtained from UCSC Xena is provided as already-normalised log_2_(norm_count+1) values rather than raw sequencing counts, the limma-trend approach (empirical-Bayes moderation with an intensity-dependent variance trend) was applied, this being the limma-recommended path for pre-normalised log-expression matrices; the classical voom mean-variance weighting, which requires raw counts, is reserved for the case where raw counts are obtained. Genes were considered significantly differentially expressed at a false discovery rate (FDR, Benjamini–Hochberg corrected [50]) below 0.05 and an absolute log_2_ fold-change of at least 1. This analysis was performed once on the full cohort (stage-agnostic DEG list) and once independently within each AJCC stage subgroup (stage-stratified DEG lists), the latter forming the candidate feature pool for the stage-stratified SHAP analysis described in Section 2.5. Because four independent stage-stratified DEG analyses multiply the overall number of statistical tests performed, FDR correction is applied both within each stage-specific analysis individually and, separately, across all stage-by-gene tests pooled together, with both results reported so that the reader can assess the multiple-testing burden under either convention (Section 2.9, Issue 4).

For full computational reproducibility, the differential-expression step is implemented in two independent codebases whose concordance is reported. The canonical analysis is performed in R using limma (https://bioconductor.org/packages/limma accessed on 11 July 2026) [26,27], and the DEG values reported in the manuscript derive from this canonical implementation. A parallel Python implementation, reproducing the same per-gene linear modelling with empirical-Bayes variance moderation, serves as an end-to-end reproducibility engine that allows the entire pipeline to be executed without R. Agreement between the two implementations (overlap of significant-gene sets and rank correlation of gene-level statistics) is quantified and reported in the Supplementary Materials as evidence of implementation-independent robustness, and all downstream analyses in the final manuscript are computed on the canonical limma gene list. The code for both implementations is released in the public repository (Section 2.8).

#### 2.4.2. Graph-Based Protein–Protein Interaction Network Analysis

DEGs from both the stage-agnostic and stage-stratified lists were mapped onto the STRING protein–protein interaction (PPI) database [28], retaining only interactions with a combined confidence score above 0.9 to ensure that only high-confidence, experimentally or computationally well-supported interactions inform network structure. Because the complete DEG list exceeds the practical input capacity of the STRING web interface (https://string-db.org, accessed on 11 July 2026), the network was constructed from the more stringently filtered DEG core (|log2FC| ≥ 2), which remains web-tractable while retaining all panel genes; the full DEG list was used for the enrichment analysis described below, where the platforms accept large gene sets. As a sensitivity analysis, the network was additionally reconstructed at a relaxed confidence threshold (0.7) to test whether the topological position of the prioritised genes was an artefact of the most stringent threshold; both analyses are reported. Densely connected sub-networks (“hub modules”) were identified using the Molecular Complex Detection (MCODE) algorithm [29] with default seeding parameters (degree cutoff = 2, node score cutoff = 0.2, k-core = 2, max depth = 100). This step is explicitly graph-based network analysis—a deterministic, parameter-light algorithm operating on a fixed graph—and not a graph neural network. A graph neural network was considered for this role and explicitly not adopted: at the sample sizes available here, a trained graph model would not be expected to outperform deterministic network analysis, and its inclusion as a token comparator would risk being read as methodological theatre rather than genuine benchmarking (Table 2).

#### 2.4.3. Triangulated Pathway Enrichment

To avoid reliance on a single enrichment database, hub genes identified by MCODE, together with the full DEG lists, were submitted in parallel to four independent enrichment platforms: DAVID (https://davidbioinformatics.nih.gov, accessed on 11 July 2026) [30], KEGG (https://www.genome.jp/kegg/, accessed on 11 July 2026) [31], Reactome (https://reactome.org, accessed on 11 July 2026) [32], and ClueGO (https://apps.cytoscape.org/apps/cluego, accessed on 11 July 2026) [33] (the latter as a Cytoscape plugin enabling network-contextualised enrichment visualisation). A pathway was considered robustly implicated only if it reached significance (FDR < 0.05) in at least two of the four platforms independently, providing convergent rather than single-source mechanistic evidence. Where a single database was queried through more than one interface (for example, KEGG via both DAVID and a dedicated enrichment service, or Reactome via both DAVID and the standalone Reactome analysis tool), the database was counted only once toward the two-of-four criterion, so that the same underlying knowledge base could not contribute multiple independent votes. The standalone Reactome service applies a conservative correction across its entire pathway hierarchy and consequently returned no terms below the significance threshold for this gene set; Reactome was therefore credited where its database reached significance through either interface, a decision documented in the released analysis code. Gene Ontology Biological Process terms returned by DAVID and ClueGO were used to characterise the biological processes represented by the DEG set and to support the gene-level interpretation in Section 2.4.4, rather than as additional independent platforms in the counting rule. We point out that the four platforms are not statistically independent, as they share a common set of pathway annotations; therefore, the two-of-four criterion is suggested as a conservative convergence heuristic to minimise dependence on any individual platform’s algorithm and annotation set, but not as a statistical validation. The per-platform enrichment statistics and their Benjamini–Hochberg FDR corrections are provided in Supplementary Table S3 to make the underlying significance clear rather than just the number of concordant enrichments.

#### 2.4.4. Two-Level Network Analysis: Locating SHAP-Prioritised Genes Within the DEG Network

The protein–protein interaction network and pathway enrichment described above are constructed on the full DEG sets (thousands of genes), a sample size at which network topology and enrichment statistics are well powered and stable. The stage-stratified SHAP-prioritised gene panel (Section 2.5.3), by contrast, is deliberately compact (of the order of tens of genes) and is too small to support robust enrichment in isolation. To connect these two scales without subjecting the small panel to underpowered standalone enrichment, a two-level analysis is performed. At the first level, the well-powered DEG network and its enriched pathways are established as above. At the second level, the SHAP-prioritised genes are located within that established network and pathway landscape: their network-topological roles are assessed (degree centrality, betweenness, and membership of MCODE hub modules), and the specific enriched pathways to which they belong are identified and reported. This design yields enrichment statistics drawn from a large, well-powered gene set while still delivering the focused, novel interpretation—whether the genes the model relies upon for its stage-dependent predictions occupy mechanistically central positions in the CRC interaction network—that links the mechanistic analysis (Contribution 3) directly to the stage-stratified explainability finding (Contribution 1). The small panel is never enriched in isolation; it is interpreted in the context of the larger network, avoiding the low-power pitfall inherent in enriching a handful of genes alone.

### 2.5. Benchmarked Classification and Stage-Stratified Explainable Machine Learning

#### 2.5.1. Six-Algorithm Benchmark

Rather than selecting a single classification algorithm a priori, six candidate models spanning linear, kernel-based, bagged-tree, boosted-tree, and shallow neural-network families were trained and compared under identical nested cross-validation folds so that the final model choice is an empirical result rather than an assumption. The six model families and their role in the comparison are summarised in Table 3.

All six models were evaluated using the same outer 5-fold stratified cross-validation split, with identical inner-loop hyperparameter-tuning budgets allocated across models so that no single algorithm benefits from a larger search. Because the tumour-versus-normal cohort is class-imbalanced (286 tumour versus 41 normal, approximately 7:1), models were screened not on AUC alone—which is insensitive to class imbalance—but on a panel of imbalance-robust metrics: the area under the precision-recall curve (AUPRC), the Matthews correlation coefficient (MCC), balanced accuracy, and per-class sensitivity and specificity, alongside AUC. To test whether imbalance materially affected model behaviour, a class-weighted variant of every model (inverse-frequency class weighting; scale_pos_weight for the gradient-boosted model) was benchmarked alongside the unweighted models. Synthetic minority oversampling (SMOTE) was deliberately not used, as interpolation in a ~16,000-dimensional expression space produces biologically implausible samples and is prone to cross-validation leakage; a measure-first, correct-only-if-needed policy was adopted instead.

Final model selection was made principled rather than ordinal: pairwise DeLong’s tests [44] on the pooled out-of-fold predictions first identified the set of models statistically indistinguishable from the top performer (*p* > 0.05); among that statistically tied set, the most parsimonious model (fewest effective parameters, greatest interpretability) was selected, consistent with Occam’s razor and favouring transparency for the downstream SHAP attribution. The explicit selection rule and the identity of the tied set are recorded in the released code as a machine-readable decision record. Uncertainty for every reported metric is quantified by 1000-iteration percentile bootstrap 95% confidence intervals (Section 2.7.2), resampling the pooled out-of-fold predictions, with resamples lacking both classes discarded so that AUC and AUPRC remain defined.

#### 2.5.2. Primary Model Training Protocol

For the empirically selected primary model, training used nested cross-validation: an outer loop of stratified 5-fold cross-validation estimates generalisation performance, while an inner loop performs hyperparameter optimisation on the corresponding inner training partition only, preventing hyperparameter selection from leaking information from the outer validation fold. Within each outer training fold, feature selection (univariate ANOVA-F pre-filtering followed by L1-penalised embedded selection) and feature standardisation are fit on the training partition alone and applied to the held-out fold without refitting so that no information from the held-out fold influences either feature choice or scaling. The hyperparameter search uses an identical, fixed per-model budget across all candidate algorithms; an equivalent Bayesian-optimisation implementation and a compact randomised-search implementation are both provided in the released code and yield the same parsimony-based selection. The empirically selected primary model was the Elastic Net logistic regression, chosen as the most parsimonious member of a statistically tied set (Section 2.5.1). All random processes—data splitting, feature selection, internal stochastic subsampling, SHAP background sampling, and bootstrap resampling—use a fixed random seed (42) to ensure the entire pipeline is exactly reproducible from the released code. The hyperparameter search space and tuning protocol for each model are detailed in Table 4.

#### 2.5.3. Stage-Stratified SHAP Attribution—Core Novel Methodological Step

SHAP (SHapley Additive exPlanations) values were computed for the empirically selected primary model using the explainer appropriate to its architecture. As the selected primary model is the Elastic Net (a linear model; Section 2.5.1), the exact LinearExplainer [23] is used, for which the Shapley attribution of each gene is exactly the product of the model coefficient and the gene’s mean-centred value; this provides exact (not approximate) Shapley attributions and satisfies the efficiency, symmetry, and dummy axioms required for an attribution method to be theoretically faithful to the underlying model. (TreeExplainer or DeepExplainer would be used analogously had a tree-ensemble or neural model been selected.) Two complementary attribution analyses were performed on the same final, locked model:

- Global attribution: mean absolute SHAP value computed across the entire training cohort, irrespective of AJCC stage, replicating the standard approach used throughout the existing CRC transcriptomic SHAP literature [13,14,15].
- Stage-stratified attribution (the core novel contribution): SHAP values computed separately within each AJCC stage subgroup (Stage I, II, III, and IV) using the same trained model and the same background distribution, yielding four independent per-stage gene-importance rankings, subject to the minimum-n-per-stage and bootstrap stability checks described next.

Given that Stage I (*n* = 45 in TCGA-COAD prior to any GEO augmentation) is the smallest subgroup, a minimum-n-per-stage threshold of 30 is pre-specified for primary reporting, and SHAP ranking stability within each stage is additionally assessed via 1000-iteration bootstrap resampling of that stage’s own samples; a stage’s ranking is reported as stable only if its top-10 gene ranking achieves Spearman ρ > 0.7 across bootstrap resamples of itself. Stages not meeting this stability bar are reported descriptively in the Results but explicitly flagged as not supporting confirmatory claims about stage-dependent gene importance (Section 2.9, Issue 2). Concordance between the global ranking and each per-stage ranking, and between pairs of stage-specific rankings, is quantified using Spearman’s rank correlation coefficient, with statistical significance assessed via permutation testing (1000 permutations of stage labels). Stage-resolved SHAP beeswarm plots, waterfall plots for representative individual patients, and dependence plots highlighting threshold effects are generated for each stage subgroup independently. In the present analysis, all four AJCC stage subgroups met both the minimum-n-per-stage threshold and the bootstrap stability bar (bootstrap mean Spearman ρ ranging from 0.86 to 0.95 across the four stages), so the four-stage analysis is reported as primary, and the early-versus-late fallback (Section 2.5.4) was not triggered; the decision outcome is recorded in the released code.

#### 2.5.4. Pre-Specified Resolution Fallback for Underpowered Stage Subgroups

It is important to emphasise that the stage-stratified SHAP analysis is a post hoc stratification of a single model trained on the full cohort (all AJCC stages combined, *n* ≈ 350–450), and not the fitting of a separate model within each small stage subgroup. No model is trained on any subgroup as small as the Stage I sample; the full-cohort model’s attributions are simply examined separately by stage. This design choice directly avoids the small-sample model-overfitting concern that arises when a predictive model is trained on a few dozen samples, because no such per-subgroup training occurs at any point.

Nonetheless, to guard against the residual risk that SHAP attributions computed on a small subgroup (notably Stage I) may be unstable, the following decision rule is pre-specified here, before any data acquisition or analysis, so that the choice of analytical resolution is a principled, pre-registered decision rather than a post hoc selection made after inspecting the results: if all four AJCC stage subgroups meet the pre-specified stability threshold (Section 2.5.3), the four-stage analysis is reported as the primary result. If one or more stage subgroups fail the stability threshold, the analysis collapses to two well-powered groups—early-stage (AJCC I–II) versus late-stage (AJCC III–IV)—and the central claim of stage-dependent gene-importance redistribution is evaluated and reported at this early-versus-late resolution instead. Under the combined cohort, the early-stage (I–II) and late-stage (III–IV) groups each contain well over one hundred samples, comfortably exceeding the stability threshold and removing the small-subgroup concern entirely at this resolution.

The outcome of applying this rule is reported explicitly in the Results regardless of which branch is taken: if the four-stage analysis succeeds, the manuscript states that the fallback was defined but not required; if the fallback is triggered, the manuscript states which subgroup(s) failed the threshold and reports the early-versus-late analysis as primary. This closed-loop reporting ensures the reader always knows both what was planned and what occurred and prevents any appearance of selectively choosing the stratification resolution that produced the most favourable result. The core scientific claim of this study—that the model’s gene-level reasoning shifts measurably with disease progression—is supported at either resolution; the four-stage analysis provides finer granularity if the data support it, while the early-versus-late analysis provides a robustly powered version of the same claim if they do not.

### 2.6. Saudi Population Contextualisation

Following SHAP-based gene prioritisation, the resulting stage-stratified gene set is cross-referenced against the actionable mutation frequencies reported for the 107-patient Saudi CRC cohort described in Section 2.2.3 [21]. This comparison is conducted exclusively at the pathway level, not at the level of individual transcript-to-mutation mapping, given that the two data types (RNA expression vs. DNA mutation) are not directly equivalent. SHAP-prioritised genes are grouped into their constituent biological pathways (as established in Section 2.4.3), and pathway membership is compared against the pathways disrupted by the high-frequency Saudi-cohort mutations (*BRCA2* and homologous recombination; *CHEK1*, *ATM*, and *ATR* and the DNA-damage-response checkpoint pathway; *PMS2* and mismatch repair). Convergence is defined as overlap in at least one enriched pathway shared between the SHAP-prioritised gene set and the genes harbouring high-frequency Saudi-cohort mutations. This study commits, in advance of running this comparison, to reporting its outcome in full regardless of direction: convergence and non-convergence will both be reported in the Results and discussed for their respective interpretive implications, eliminating the risk of this comparison being selectively reported only if favourable (Section 2.9, Issue 5). This comparison is explicitly not statistical validation, external validation, or replication in a Saudi sample, since no Saudi transcriptomic data of any kind are used in model training, tuning, or testing at any stage of this study. To be explicit about scope, “Saudi population contextualisation” here denotes hypothesis-generating biological concordance at the pathway level only: it is neither population-level validation nor transcriptomic validation in Saudi patients. No Saudi individual-level, sequencing, or expression data entered model training or validation at any point.

#### 2.6.1. Literature-Based Corroboration of Prioritised Genes

Because prospective wet-laboratory validation (for example, qRT-PCR or immunohistochemistry of the prioritised genes in fresh patient samples) is outside the scope of a computational study, but the absence of any biological corroboration would be a genuine weakness rather than an unavoidable one, each SHAP-prioritised gene is systematically cross-referenced against the existing experimental literature. For every gene in the final panel, a structured search records whether prior studies report experimental (qRT-PCR, Western blot, immunohistochemistry, or functional) evidence of its dysregulation or mechanistic role in colorectal cancer, and this evidence is tabulated alongside the computational findings. This step does not substitute for prospective experimental validation, but it establishes whether the computationally prioritised genes are biologically plausible and already supported by independent wet-lab evidence, distinguishing genuinely novel candidates from those with established experimental backing. Genes with no prior experimental evidence are explicitly flagged as novel, hypothesis-generating candidates warranting future laboratory validation.

#### 2.6.2. Expression-Direction Confirmation in an Independent Transcriptomic Cohort

As an additional, explicitly supportive plausibility check, the tumour-versus-normal expression direction (up- or down-regulation) of each SHAP-prioritised gene is confirmed in an independent transcriptomic resource not used in model development—principally GEPIA2 (http://gepia2.cancer-pku.cn, accessed on 11 July 2026), which serves uniformly reprocessed TCGA and GTEx expression data, with a further independent GEO cohort consulted where available. This check tests only whether the direction of differential expression observed during discovery is reproducible in an independently assembled expression dataset, thereby guarding against the possibility that a prioritised gene’s signal is an artefact of the primary cohort. It is stated explicitly that this is not qRT-PCR and is not an orthogonal, wet-laboratory validation: it remains within the transcriptomic modality and therefore constitutes internal-consistency support rather than independent-method confirmation. It is reported as such and is never described using wet-laboratory terminology.

The reporting weight of this check is fixed in advance, independent of its outcome, to avoid outcome-dependent emphasis. Its detailed results are reported in the Supplementary Materials in all cases. Its outcome is discussed in the main-text Discussion regardless of direction—a confirmed expression direction is reported as supporting plausibility, and a non-confirmed direction is reported as a caveat that readers require—mirroring the pre-commitment applied to the Saudi pathway comparison (Section 2.6). This check is deliberately excluded from the Abstract irrespective of how favourable its result proves to be, because it is a secondary supporting detail rather than a primary finding, and the Abstract is reserved for the central stage-stratified attribution result, primary model performance, and primary external validation. This allocation is determined by the check’s subordinate scientific role, not by the favourability of its result.

### 2.7. Survival Modelling and External Validation

#### 2.7.1. Benchmarked Survival Architectures

Three survival modelling approaches were benchmarked on the SHAP-prioritised gene panel, using overall survival time and censoring status from TCGA-COAD clinical annotation as the outcome:

- Cox proportional hazards (Cox PH) regression—the conventional baseline, assuming a linear, time-invariant effect of each covariate on the log-hazard.
- DeepSurv [39]—a deep neural-network extension of the Cox model that learns non-linear covariate interactions, included as the deep-learning comparator and intentionally benchmarked rather than assumed superior.
- Random Survival Forest (RSF) [40]—an ensemble tree-based survival method that estimates the cumulative hazard non-parametrically, included as a non-deep-learning, non-parametric comparator.

Per the events-per-variable resolution adopted for this study [46], stage-specific event counts are tabulated in the Results before any stage-stratified survival benchmarking is reported; stages meeting the conventional events-per-variable (EPV) ≥ 10 threshold are reported as confirmatory, and stages below this threshold are explicitly labelled exploratory rather than omitted so that the reader can judge the evidentiary weight of each stage’s survival findings directly. All three models were trained on the same SHAP-prioritised feature set under the same cross-validation folds described in Section 2.5, with hyperparameters for DeepSurv (hidden layer sizes, dropout, and learning rate) and RSF (number of trees and minimum leaf size) each tuned via the same nested Bayesian optimisation protocol used for the primary classifier.

#### 2.7.2. Performance Metrics and External Validation

Model discrimination was quantified using Harrell’s concordance index (C-index) [43] and time-dependent area under the receiver–operating–characteristic curve (AUC) evaluated at 3-year and 5-year horizons, the two time points most commonly reported in comparable CRC survival literature and most clinically relevant for treatment-planning decisions. All three models and all six classification-stage benchmark models (Section 2.5.1) were evaluated on GSE39582 and GSE17536/GSE17537 [47], the open-access external validation cohorts adopted in place of ICGC following the access-tier change documented in Section 2.2.2 and Section 2.9 (Issue 6); none of these cohorts was used during any stage of model development.

Pairwise comparison of AUC values between models was performed using DeLong’s test for correlated ROC curves [44], which is now expected by reviewers as the minimum standard for comparing model discrimination rather than reporting point AUC estimates alone. Uncertainty in all primary performance metrics (C-index, time-dependent AUC, sensitivity, specificity) was quantified using 1000-iteration bootstrap resampling, with 95% confidence intervals computed via the percentile method. All reporting follows the TRIPOD + AI guideline for clinical prediction models using regression or machine learning methods [22], and a completed TRIPOD+AI checklist is provided as a supplementary file at submission.

### 2.8. Statistical Software and Reproducibility

All analyses were conducted in Python 3.10 (scikit-learn 1.3.2, XGBoost 2.0.3 [12], LightGBM 4.3.0 [37], SHAP 0.44.1 [23], scikit-survival 0.22.2, lifelines 0.28.0, Optuna 3.6.1 [51], GEOparse 2.0.4, pyComBat 0.3.3) and R 4.3 (limma 3.58.1 [26,27], STRINGdb 2.14.3 [28], MCODE 2.0.3 [29] via Cytoscape 3.10.2 (Institute for Systems Biology, Seattle, WA, USA)), with exact package versions pinned in a published requirements.txt and renv.lock file. The complete analysis pipeline, from raw data download through to final figure generation, is released in a public GitHub repository under an MIT licence, accompanied by Google Colab notebooks (https://colab.research.google.com, accessed on 11 October 2026) that reproduce every reported result without requiring local installation. Prior to public release, the repository is reviewed by co-author Sultan Alhayyani (Section 2.9, Issue 8). A bioRxiv preprint will be posted concurrently with journal submission. All random seeds are fixed at 42 throughout, and this value, together with the exact software versions and hardware specifications used, is documented in the repository README to support independent replication.

### 2.9. Methodological Risks Identified Prior to Data Acquisition and Their Resolutions

Eight methodological risks were identified during planning and resolved, in writing, before any data acquisition began, so that each represents a pre-registered decision rather than a post hoc justification. Table 5 summarises each risk and the resolution adopted; the corresponding resolution is also cross-referenced at the relevant point earlier in this section.

### 2.10. Audited Study Limitations and Their Doability Status

Every limitation of this study was audited against a single explicit test before being accepted as a limitation: is it actually fixable within the scope of a computational study using public data? A limitation is only legitimately declared as such if the answer is no, or if the doable portion has already been performed and only an inherent residual remains. This standard prevents the use of a limitations statement as a shield for avoidable gaps. Table 6 records the outcome of this audit. Two limitations that an earlier draft would have simply declared—external stage-stratified validation and biological corroboration of prioritised genes—were found to be partly doable; the doable portions have been incorporated into the methodology above (Section 2.2.2 and Section 2.6.1, respectively), and only the genuinely unavoidable residuals are carried forward to the manuscript’s Limitations section (Section 4.6). This audited inventory is provided here so that the eventual Limitations and Future Work sections report only constraints that survive the doability test, paired where appropriate with the future work that would resolve them.

## 3. Results

### 3.1. Differential Expression Reveals Extensive, Reproducible Transcriptomic Reprogramming

**Figure 2:**
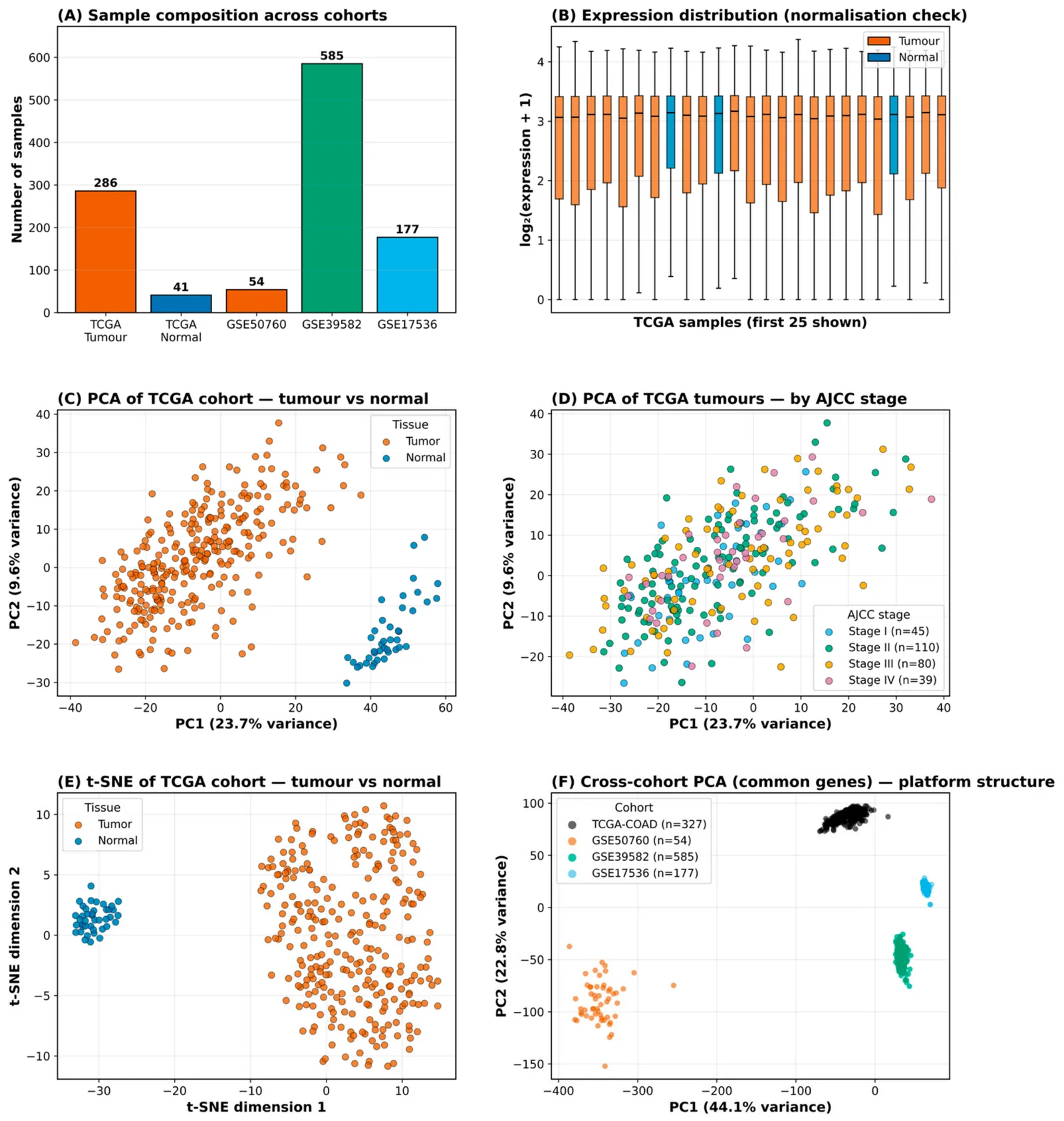
Quality-control overview of the discovery cohort. (**A**) Sample composition across the discovery and validation cohorts (tumour, normal, and per-stage counts). (**B**) Per-sample expression-distribution boxplots for the TCGA cohort, confirming consistent normalisation and comparable library sizes. (**C**) Principal component analysis (PCA) of the TCGA cohort coloured by tumour versus normal. (**D**) PCA of the TCGA tumours coloured by AJCC stage. (**E**) t-SNE projection of the TCGA cohort coloured by tumour versus normal. (**F**) Cross-cohort PCA on the common genes, showing platform-related structure between cohorts.

**Figure 3:**
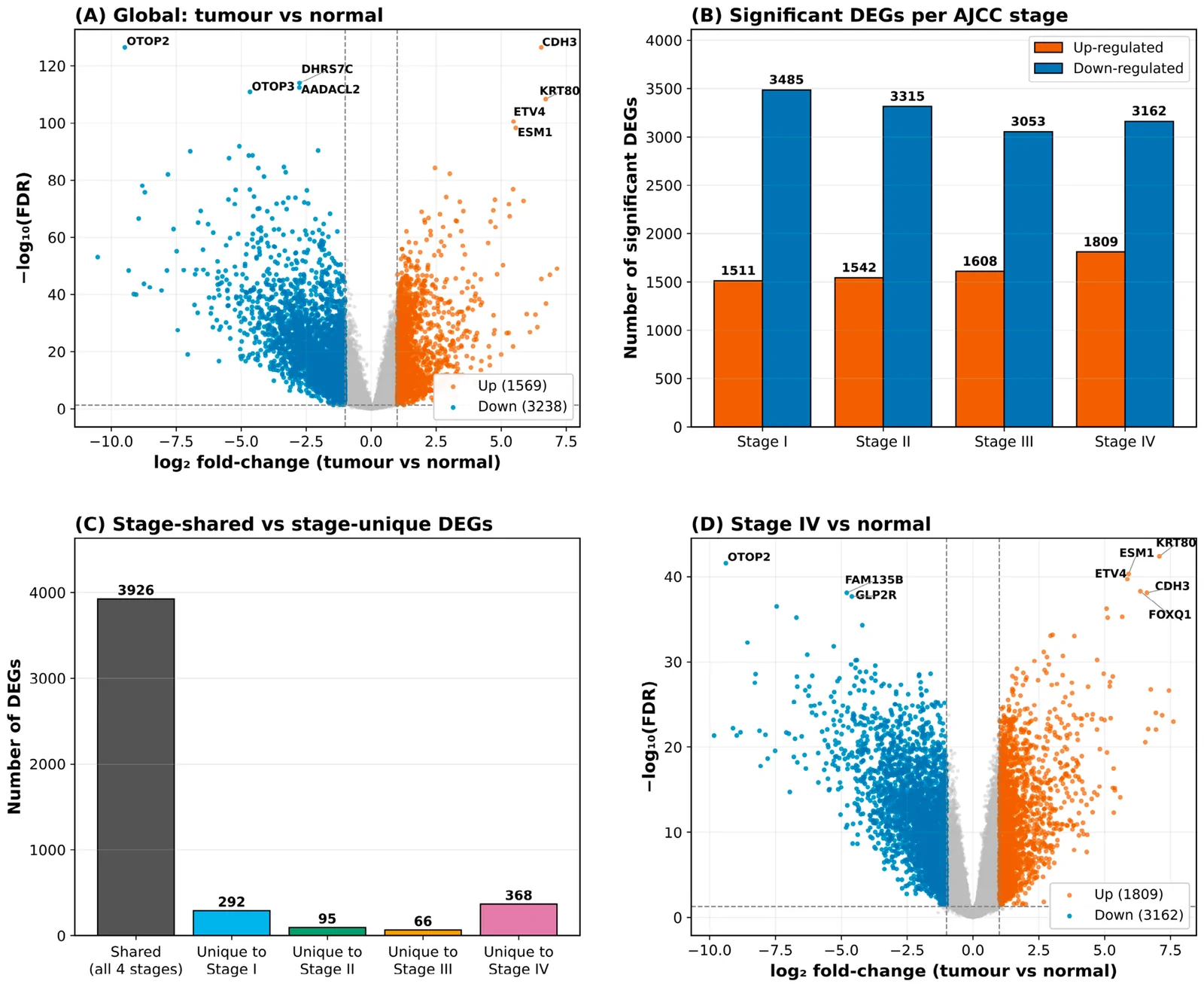
Differential-expression landscape for the tumour-versus-normal contrast. (**A**) Global volcano plot across all tumour samples, plotting log2 fold-change against statistical significance (−log10 FDR), with the panel genes highlighted and labelled. (**B**) Number of significantly up- and down-regulated genes identified at each AJCC stage. (**C**) Number of DEGs shared across all four stages versus those unique to a single stage. (**D**) Volcano plot for the Stage IV versus normal contrast, with the panel genes highlighted and labelled. In the volcano panels (**A**,**D**), dashed lines mark the significance thresholds (|log2 fold-change| = 1 and FDR = 0.05); grey points denote genes that did not meet the significance threshold (FDR ≥ 0.05 or |log2 fold-change| < 1), while orange and blue points denote significantly up- and down-regulated genes, respectively.

Following quality control of the discovery cohort (Figure 2), canonical limma analysis [26,27] of the discovery cohort identified 4807 differentially expressed genes (DEGs; 1569 up-regulated, 3238 down-regulated) at FDR < 0.05 and |log2FC| ≥ 1 (Figure 3). An independent Python re-implementation reproduced this set almost exactly (Jaccard 0.995; Spearman correlation of log2FC = 1.000; 100% direction agreement across all contrasts; Table S1), so the differential-expression layer is implementation-independent. Comparable DEG counts at every AJCC stage (4682–4979; Table S1) indicate that substantial reprogramming is established already at Stage I rather than accruing gradually, motivating the stage-resolved interpretation that follows.

### 3.2. A Compact Ten-Gene Panel Classifies Tumour Versus Normal, Robustly to Class Imbalance

**Figure 4:**
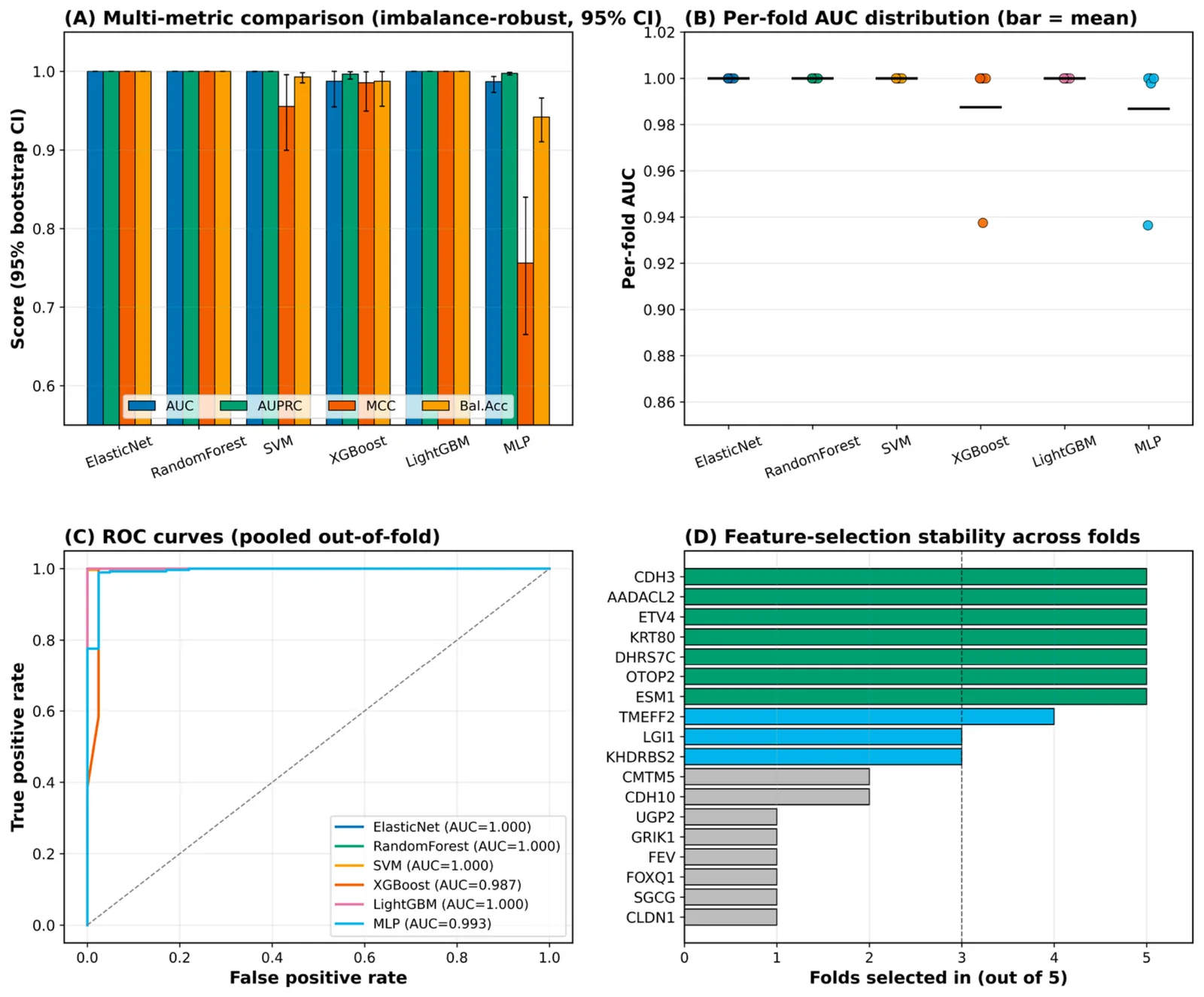
Cross-validated performance of the six classifiers. (**A**) Multi-metric comparison across discrimination and imbalance-robust metrics (AUC, AUPRC, MCC, balanced accuracy), with 1000-iteration bootstrap 95% confidence intervals shown as error bars. (**B**) Distribution of per-fold AUC for each classifier (bar = mean). (**C**) Pooled out-of-fold ROC curves, with the area under the curve (AUC) given for each classifier; classifiers attaining AUC = 1.000 (Elastic Net, Random Forest, SVM, LightGBM) produce overlapping ROC curves along the upper-left axis, so only XGBoost (AUC = 0.987) and MLP (AUC = 0.993) are separable. (**D**) Feature-selection stability across the five cross-validation folds, showing the number of folds (out of five) in which each gene was selected.

Cross-validated stability selection converged on a ten-gene panel (*CDH3*, *OTOP2*, *DHRS7C*, *AADACL2*, *KRT80*, *ETV4*, *ESM1*, *TMEFF2*, *LGI1*, *KHDRBS2*; seven genes recurring in all five folds). Differential-expression statistics for all ten panel genes are given in Table S2. Six classifiers were benchmarked under a leakage-free protocol with 1000-iteration bootstrap confidence intervals (CIs) (Figure 4). The Elastic Net, Random Forest and LightGBM each reached an internal AUC of 1.000 (95% CI 1.000–1.000); because all leading models were statistically indistinguishable by the DeLong test (pairwise *p* > 0.05), the Elastic Net was carried forward as the most parsimonious and interpretable member of the tied set. The perfect internal discrimination reflects the large transcriptomic separation between tumour and adjacent-normal tissue rather than model overfitting: it was obtained under a strictly leakage-free protocol in which all feature selection and preprocessing were confined to training folds, was reproduced by three independent algorithms, and—most importantly—was corroborated by strong discrimination in a fully external cohort (AUC 0.969), where overfitting to the discovery data could not contribute. Because near-perfect internal discrimination warrants scrutiny, we note it reflects the large transcriptomic distance between tumour and adjacent-normal tissue rather than overfitting: it was obtained under a strictly leakage-free protocol, reproduced by three independent algorithm families, and corroborated by strong discrimination in a fully external cohort (AUC 0.969) where overfitting to the discovery data cannot contribute; AUPRC remained near-maximal and class weighting produced negligible change (Table 7). A 1000-iteration permutation (label-shuffling) test confirmed that the observed AUC lies far beyond the permuted-label null (null mean 0.50, maximum 0.69; permutation *p* < 0.001), and repeated nested cross-validation across ten seeds was invariant (mean AUC 1.000, SD 0.000) (Supplementary Table S10, Figure S4).

Because the cohort is imbalanced (286 tumour versus 41 normal, ≈7:1), a dedicated analysis established that this imbalance did not distort model behaviour—a question of sufficient importance that it was tested directly rather than assumed (Table 7). Three lines of evidence converge. First, the imbalance-robust area under the precision–recall curve (AUPRC) remained at or near 1.000 for every leading model, demonstrating that performance was not an artefact of the majority class. Second, inverse-frequency class weighting produced no material change in any metric (for the Elastic Net, Matthews correlation coefficient [MCC] 1.000 unweighted versus 0.988 weighted; balanced accuracy 1.000 versus 0.998), indicating the decision boundary was already well calibrated to both classes. Third, synthetic minority oversampling (SMOTE) was deliberately avoided: interpolation within a high-dimensional expression space risks generating biologically implausible profiles and cross-validation leakage, and the preceding two results showed it to be unnecessary. The panel therefore classifies reliably on both classes rather than defaulting to the majority.

### 3.3. Feature-Importance Structure Shifts Measurably and Monotonically Across Stages

**Figure 5:**
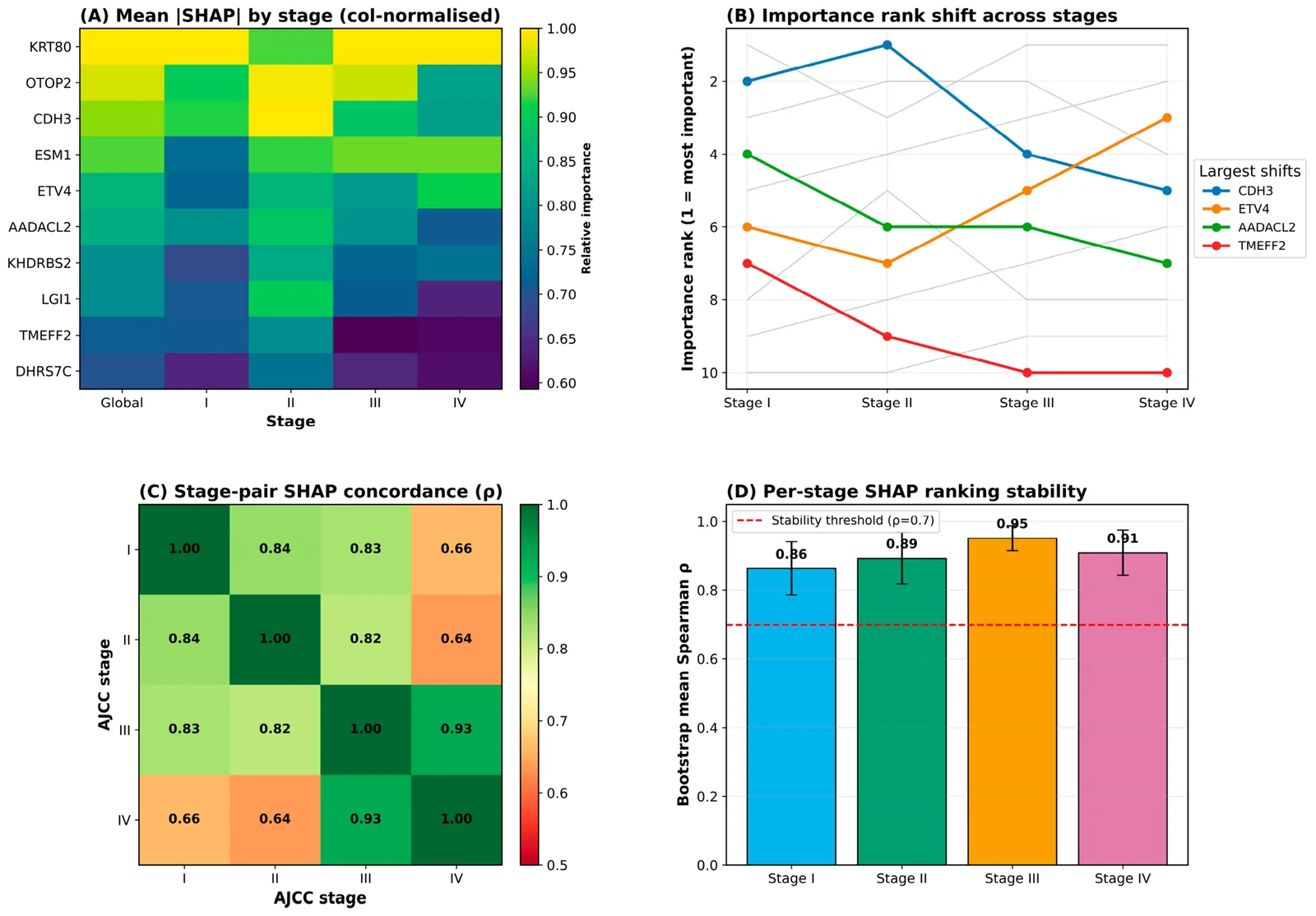
Stage-resolved SHAP attribution across AJCC stages. (**A**) Column-normalised heatmap of the mean |SHAP| value for each panel gene, shown for the global model and for each stage (I to IV), so that relative within-stage importance can be compared across stages. (**B**) Importance-rank trajectories of the panel genes from Stage I to Stage IV (rank 1 = most important); the thin grey lines show the stage-to-stage rank trajectories of the remaining panel genes, and the genes with the largest rank shifts are highlighted in colour. (**C**) Stage-pair concordance of the SHAP-based importance rankings, expressed as the Spearman correlation (ρ) between every pair of stages. (**D**) Per-stage stability of the SHAP importance ranking, shown as the bootstrap mean Spearman ρ with error bars; the dashed line marks the stability threshold (ρ = 0.7).

Computing exact linear SHAP attributions [23] within each AJCC stage revealed that, although the panel classifies tumours at every stage, the internal ranking of feature importance reorganises as disease advances (Figure 5). Each stage-specific profile was individually stable under bootstrap resampling (mean Spearman ρ 0.86–0.95; all ρ > 0.7), supporting a four-stage rather than a coarser analysis. A monotonic concordance gradient was observed: the extreme stages were least alike (Stage I versus IV, ρ = 0.661, *p* = 0.038), whereas adjacent late stages were highly concordant (Stage III versus IV, ρ = 0.927). Every stage profile also differed significantly from the stage-agnostic global ranking (all *p* < 0.005), confirming that stratification recovers information a pooled model discards. The dominant shift was the ascent of *ESM1* from mid-importance early to a leading late-stage position. Stage-wise bootstrap 95% confidence intervals for these concordance statistics are reported in Supplementary Table S8 (for example, Stage I vs. IV rho = 0.661, 95% CI [0.074, 0.923]; Stage III vs. IV rho = 0.927, 95% CI [0.617, 1.000]); the smallest subgroup (Stage IV, *n* = 39) motivated both the bootstrap stability check and the pre-registered early- versus late-stage fallback (Section 2.5.4), ensuring the reported patterns are not driven by a few influential samples.

### 3.4. The Stage-Dependent Importance Shift Replicates in an Independent Cohort

**Figure 6:**
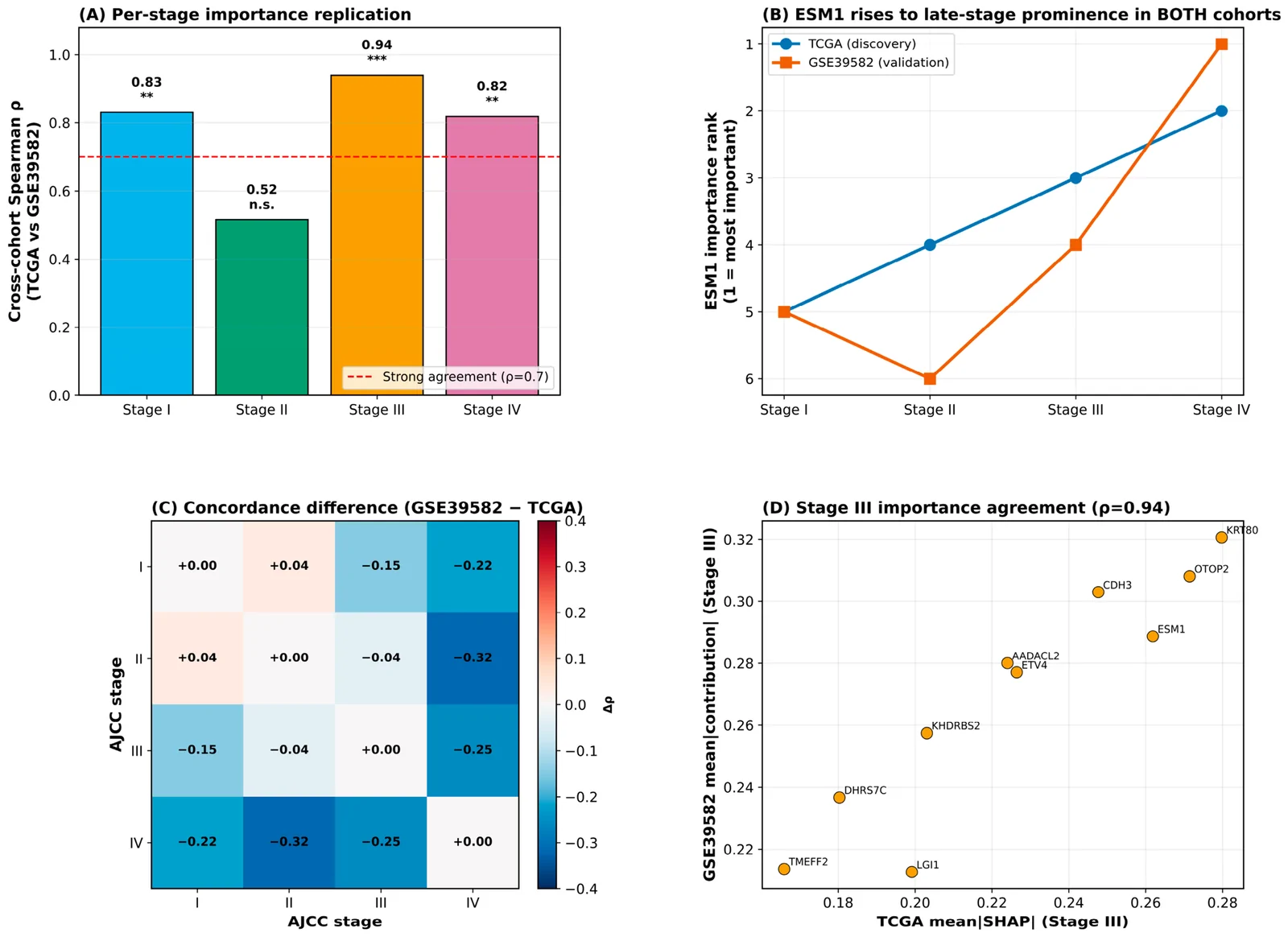
Cross-cohort replication of stage-resolved feature importance between the discovery cohort (TCGA) and the external validation cohort (GSE39582). (**A**) Per-stage replication of the importance rankings, shown as the cross-cohort Spearman correlation (ρ) between TCGA and GSE39582 at each stage; the dashed line marks strong agreement (ρ = 0.7). ** *p* < 0.01; *** *p* < 0.001; n.s., not significant (significance of the cross-cohort Spearman correlation). (**B**) Importance rank of *ESM1* across stages in both cohorts (rank 1 = most important), showing its rise to late-stage prominence. (**C**) Concordance difference between stage pairs (GSE39582 − TCGA). (**D**) Agreement of per-gene importance at Stage III (ρ = 0.94), plotting TCGA mean |SHAP| against the corresponding GSE39582 importance for each panel gene.

Transferring the exact attribution to the independent GSE39582 cohort (*n* = 579, full AJCC staging) tested whether this structure was cohort-specific (Figure 6). Per-stage importance concordance was significant at three of four stages (Stage I ρ = 0.83; Stage III ρ = 0.94; Stage IV ρ = 0.82; all *p* ≤ 0.004), with Stage II not reaching significance (ρ = 0.52, *p* = 0.13). Crucially, the late-stage prominence of *ESM1* replicated, reaching top-two importance at Stage IV in both cohorts, whereas the TCGA-specific rise in *ETV4* did not—demonstrating that the framework distinguishes reproducible stage-dependent drivers from cohort-specific signals rather than asserting uniform replication.

### 3.5. Prognostic Assessment Defines the Signature’s Scope

**Figure 7:**
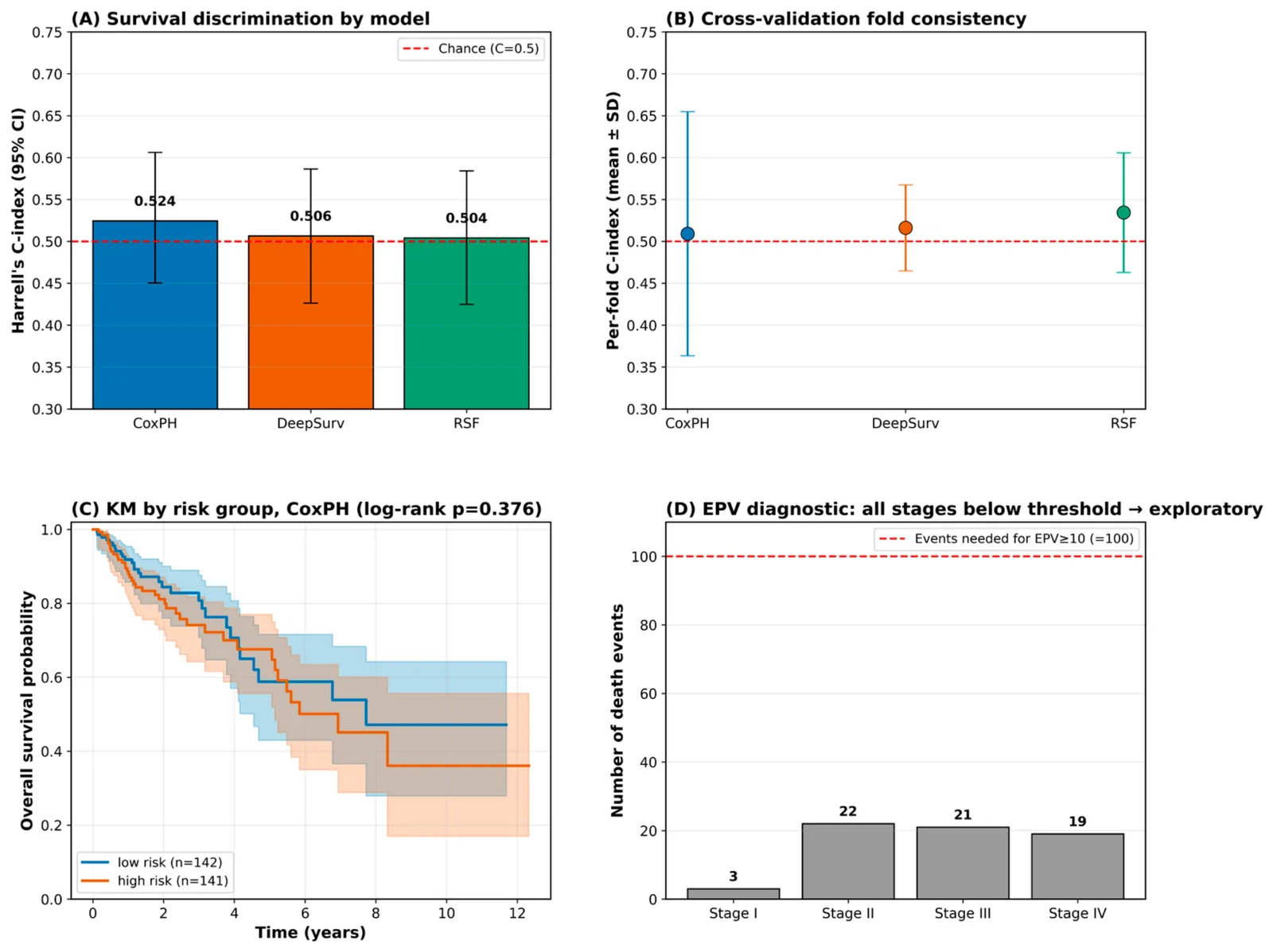
Survival-model benchmarking on the ten-gene panel. (**A**) Survival discrimination by model, shown as Harrell’s concordance index (C-index) with 95% bootstrap confidence intervals; the dashed line marks chance (C-index = 0.5). (**B**) Cross-validation fold consistency, showing the per-fold C-index distribution (strip plot with mean) for each model. (**C**) Kaplan–Meier survival curves for the panel-defined risk strata (best model, median split), with the log-rank *p* value. (**D**) Events-per-variable (EPV) diagnostic, showing the event count per stage against the EPV ≥ 10 threshold; all stages fall below it, confirming the survival analysis is exploratory.

The panel’s prognostic value for overall survival was evaluated against the pre-registered events-per-variable (EPV) criterion [46] (Figure 7). All per-stage subgroups fell below EPV ≥ 10 and were designated exploratory in advance. Across Cox proportional hazards, DeepSurv and random survival forest models, discrimination was indistinguishable from chance (C-index 0.525, 0.506 and 0.504, respectively; every CI spanning 0.5), and Kaplan–Meier stratification showed no separation (log-rank *p* = 0.376). Rather than a shortcoming, this result defines the signature’s operating scope: the panel was selected for, and excels at, tumour-versus-normal classification, and is explicitly not advanced as a prognostic model.

### 3.6. External Validation Confirms a Generalisable Classifier of Defined Scope

**Figure 8:**
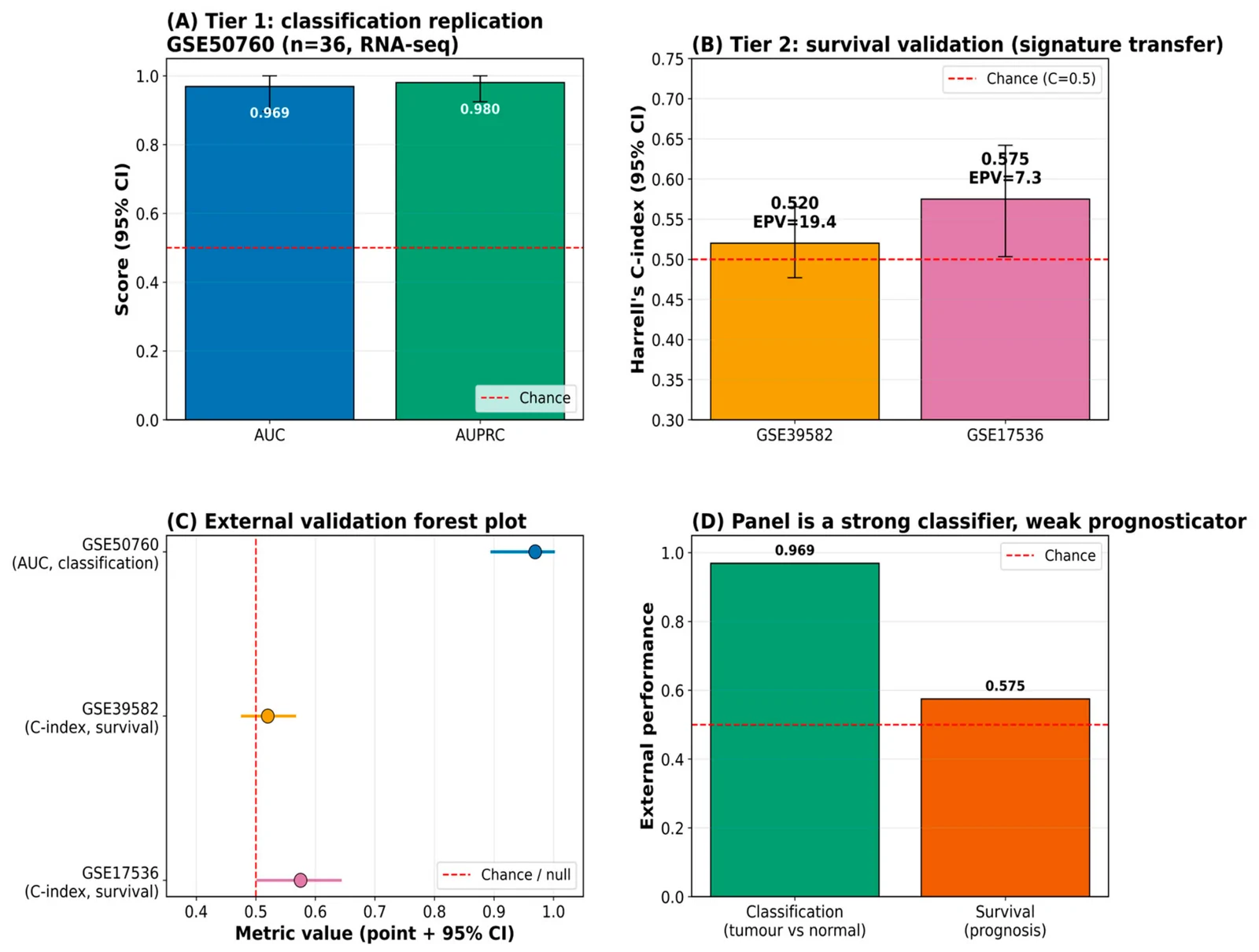
Three-tier external validation of the ten-gene panel. (**A**) Tier 1 classification replication in the independent RNA-seq cohort (GSE50760), shown as AUC and AUPRC with bootstrap confidence intervals. (**B**) Tier 2 survival validation (signature transfer), shown as the concordance index (C-index) for each microarray cohort with bootstrap confidence intervals; the dashed line marks chance (C-index = 0.5). (**C**) Forest plot summarising the point estimates and 95% confidence intervals across all validation tiers. (**D**) Summary contrast showing that the panel is a strong classifier but a weak prognosticator.

Three independent cohorts established the signature’s external behaviour (Figure 8). As a classifier it generalised strongly: in the independent RNA-seq cohort GSE50760 the external AUC was 0.969 (95% CI 0.896–1.000; area under the precision-recall curve [AUPRC] 0.980), confirming that the tumour-versus-normal signal transfers to data generated by a different laboratory. Survival validation, by contrast, reaffirmed the null prognostic finding in the only adequately powered cohort (GSE39582, EPV 19.4; C-index 0.520, 95% CI 0.477–0.565). The convergent evidence delineates the scope precisely: a reproducible classifier (external AUC ≈ 0.97) that does not function as a prognostic model. Performance is reported per cohort rather than pooled, reflecting their distinct roles and differing platform, size, and clinical composition; a consolidated cohort characteristics and per-cohort performance summary is provided in Supplementary Table S9. We note that GSE50760 is a modest cohort (18 tumour, 18 normal, metastatic disease represented), so its external AUC estimate carries a correspondingly wide confidence interval and is interpreted as supportive rather than definitive.

### 3.7. The Panel Recovers Established Biology and Nominates Novel Candidates

Biological corroboration was sought at three independent levels (Table S7). Six of the ten genes have direct experimental support in CRC, each matching the differential-expression direction recovered here: *KRT80*, previously reported as the most up-regulated gene in TCGA CRC [52]; the down-regulated tumour suppressor *OTOP2* [53]; the over-expressed, prognosis-associated *CDH3* [54]; the angiogenesis driver *ESM1* [55,56]; the pro-metastatic transcription factor *ETV4* [57]; and the methylation-silenced suppressor *TMEFF2*. Independent GEPIA2 analysis confirmed the expression direction of all ten genes, six reaching the platform’s significance threshold (Figure S3). The remaining four genes (*AADACL2*, *KHDRBS2*, *LGI1*, and *DHRS7C*) are poorly characterised in CRC and are advanced as hypothesis-generating candidates—the panel thus simultaneously recovers known biology, which validates the unbiased pipeline, and nominates new candidates.

### 3.8. The Machine-Learning Signature Is Complementary to Classical Network Hubs

**Figure 9:**
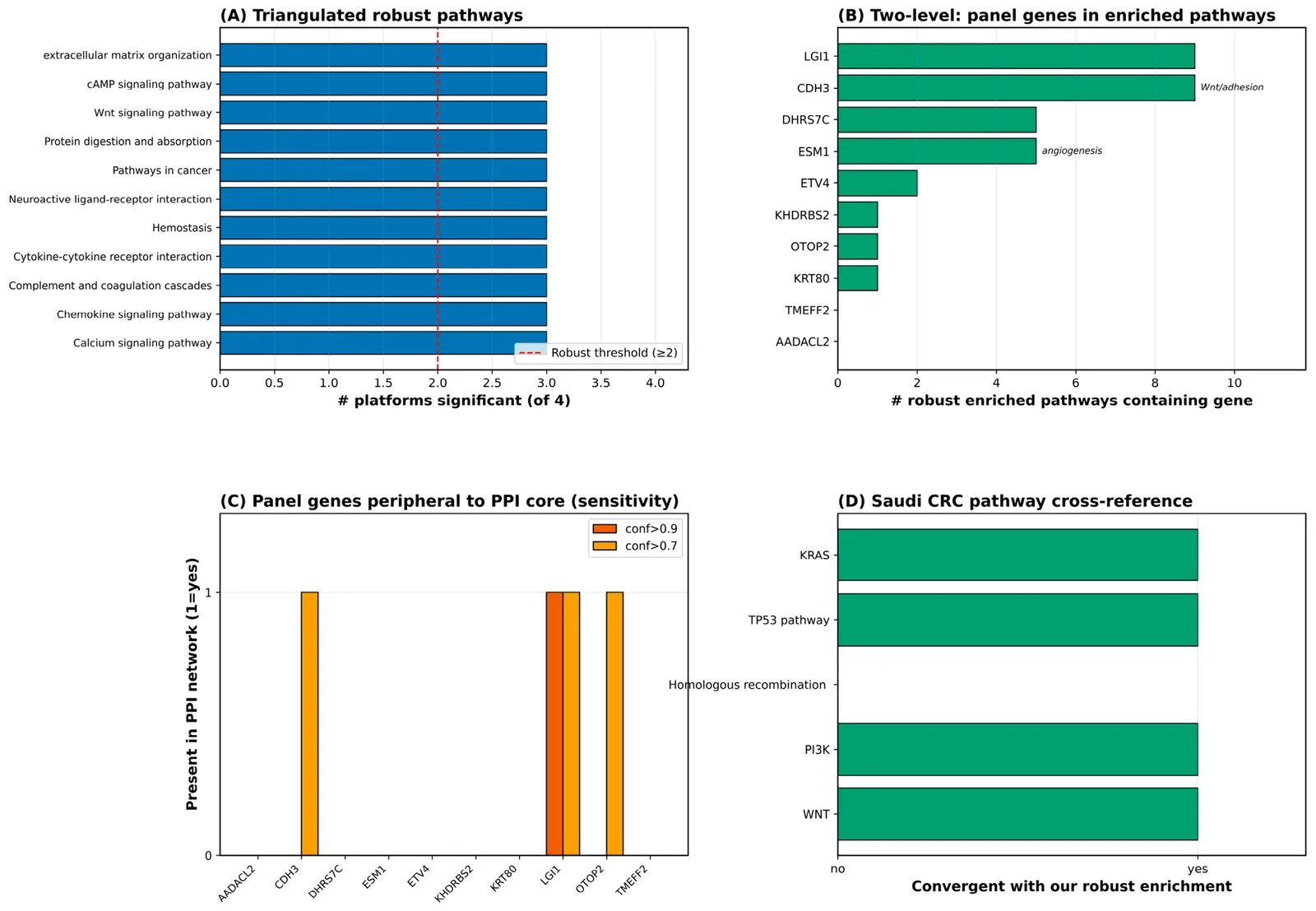
Integrated network and enrichment summary. (**A**) Robust pathways ranked by the number of significant platforms (of four). (**B**) Number of robust enriched pathways containing each panel gene. (**C**) Panel-gene presence in the PPI network at two confidence thresholds (conf > 0.7 and conf > 0.9). (**D**) Convergence of the robust pathways with reported Saudi colorectal-cancer alterations. Throughout, “#” denotes “number of.”

Triangulated enrichment of the full DEG set across four platforms identified 216 robustly enriched pathways (significant in ≥2 of 4; 55 in ≥3 of 4), dominated by canonical CRC processes including Wnt signalling, cytokine and calcium signalling, extracellular-matrix organisation and cell adhesion (Figure 9; Table S3). A two-level analysis then localised the panel within both the protein–protein interaction (PPI) network and this pathway landscape—and revealed an informative dissociation. The panel genes were largely absent from the high-confidence PPI core (one of ten present at confidence >0.9; three of ten at >0.7, none interconnected; Table S4) and belonged to none of the 32 MCODE hub modules, yet eight of ten resided in robustly enriched pathways (Table S5), with *CDH3* in Wnt and adhesion pathways and *ESM1* in angiogenesis. The signature is therefore embedded in cancer biology at the pathway level while being peripheral at the interaction-topology level, establishing that the explainable-AI panel is complementary to—not a rediscovery of—classical hub-gene analysis and would have been missed by a connectivity-based pipeline.

### 3.9. Enriched Pathways Converge with the Saudi Colorectal-Cancer Landscape

To contextualise the findings for the Saudi population without using Saudi data for training or validation, the robust pathways were cross-referenced against published Saudi CRC molecular features (Table S6). Four of five frequently altered Saudi pathways converged with the robust enrichment set: Wnt/β-catenin (altered in 65% of Saudi cases [58]), *PI3K* (48% [58]), *TP53* (~50% [21]), and *KRAS*/RAS–MAPK signalling; the single non-convergence, homologous recombination (61% [58]), is reported transparently. The Wnt axis is simultaneously the most frequently altered Saudi pathway, a robust pathway here, and the pathway containing the panel gene *CDH3*—providing a direct, population-relevant link for the *CDH3*/Wnt axis and a concrete anchor for future Saudi-specific validation.

## 4. Discussion

Most transcriptomic classifiers for colorectal cancer report a performance figure and a gene list, treating the disease as a static entity and leaving unexamined whether the model’s internal reasoning is stable across the clinical stages over which the disease actually evolves. The present study reframes the question. Using a stage-stratified explainable-AI pipeline, we show that the feature-importance structure of a reproducible transcriptomic classifier is itself stage-dependent and externally replicable, and we delineate, rather than overstate, the resulting signature’s scope. Three contributions distinguish this work from the prevailing literature, and we discuss each in turn before addressing limitations and future directions.

### 4.1. A Shift from Explaining Predictions to Tracking How Explanations Change with Stage

The principal novelty is conceptual. Explainable-AI studies in oncology typically apply SHAP or comparable methods once, to a single pooled model, and report which genes matter on average. We instead compute exact attributions within each disease stage and ask whether the importance structure itself moves. It does: a stable, monotonic gradient separates early- from late-stage reasoning (Section 3.3), and—the decisive test—this gradient replicates in an independent cohort (Section 3.4). To our knowledge, this is the first demonstration in CRC that explainability output is not a static description of a model but a stage-resolved, reproducible biological signal in its own right. The practical consequence is that interpretability becomes a substrate for discovery rather than a post hoc justification: the late-stage ascent of *ESM1* was surfaced not by differential expression (where it is one of thousands of significant genes) but by the movement of its attribution across stages, and it is precisely this gene that carries the strongest independent mechanistic support. This distinction is what separates the present work from prior SHAP-based CRC studies: earlier models report a single pooled importance ranking on comparable data [13,14,15], and the only study to demonstrate a stage-dependent importance shift did so using clinical and demographic covariates rather than gene expression [16]; here the shift is established at the transcriptomic level and shown to replicate across cohorts, which no previous CRC study has reported. We emphasise that SHAP quantifies each gene’s contribution to the model’s prediction rather than its causal or regulatory role in tumour biology; accordingly, a rise in a gene’s importance at a later stage denotes increased predictive salience within the model and should not be read, on its own, as a demonstrated increase in biological contribution. Equally, the panel is a tumour-versus-normal classifier and the stage-stratified analysis characterises how its explanation structure changes across AJCC stages; it is not proposed as an anatomical re-staging tool, and translating this finding into a clinically actionable staging aid would require purpose-built, stage-discriminative modelling and prospective evaluation [59].

### 4.2. ESM1 as a Reproducible, Mechanistically Supported Late-Stage Driver

The convergence of evidence on *ESM1* (endocan) is the study’s most biologically coherent finding. Its late-stage importance replicates across cohorts (Section 3.4); it resides in the angiogenesis pathway in our enrichment (Section 3.8); and experimental literature establishes that *ESM1* promotes CRC angiogenesis through the PI3K/Akt/mTOR axis [55] and that elevated endocan marks deeper invasion, lymph-node metastasis and advanced TNM stage [56]. Three independent computational observations therefore align with a known mechanism, positioning *ESM1* as a candidate marker of the angiogenic switch of advanced CRC. Because endocan is a secreted, serum-detectable glycoprotein [56], its stage-dependence is translationally attractive and motivates a focused, minimally invasive validation, which we identify below as a priority. Two cautions apply to this interpretation. First, because bulk transcriptomic data cannot resolve cellular composition, stage-associated differences in stromal content, immune infiltration, or tumour purity are potential confounders of the observed attribution shifts, and a partial-correlation test showed that the *ESM1*-stage association retains significance after adjustment for the other nine panel genes (partial rho = 0.120, *p* = 0.045; Supplementary Table S11), indicating an independent stage-associated component, although the modest effect size and the need for full tumour-microenvironment deconvolution (requiring cell-type data not available for this bulk cohort) temper any causal reading. Second, the increased late-stage SHAP importance of *ESM1* reflects greater predictive salience in advanced-stage models and may track the broader advanced-stage transcriptional programme rather than an independent, stage-specific determinant; *ESM1* is therefore advanced as a reproducible, hypothesis-generating signal rather than a validated target, a distinction underscored by recent work separating computationally prioritised candidates from experimentally validated therapeutic relevance [60].

### 4.3. Machine-Learning Importance and Network Centrality Capture Complementary Biology

The second novel finding concerns the relationship between explainable-AI signatures and classical network biology. Hub-gene pipelines, which dominate transcriptomic biomarker discovery, prioritise highly connected nodes. Our panel is the opposite: peripheral to the high-confidence PPI core and absent from every detected hub module, yet embedded in robustly enriched cancer pathways (Section 3.8). This dissociation is robust to network stringency (it holds at both confidence 0.9 and 0.7) and carries a clear methodological message—discriminative importance and topological centrality are complementary, not interchangeable, axes of relevance. A connectivity-based pipeline would not have recovered this signature, and treating hub centrality as a proxy for biomarker utility would have discarded genes of demonstrable discriminative and, for *ESM1*, mechanistic value. This argues for pairing, rather than substituting, explainable-AI feature selection with network analysis.

### 4.4. Interpretability, Transparent Scope Delineation, and Trustworthy AI

The third contribution is methodological and is inseparable from the choice of an interpretable model. By selecting a linear Elastic Net from a statistically tied set, every attribution in this study is an exact Shapley value—the closed-form product of a model coefficient and a standardised expression value—rather than an approximation, which is what makes the cross-cohort transfer of attributions mathematically well defined and the stage-resolved importance maps trustworthy rather than indicative. This interpretability is not decorative: it is the mechanism by which the stage-dependent signal (Section 4.1) is both computed and externally tested. Coupled with interpretability is an equally deliberate commitment to scope. Through a pre-registered EPV criterion and three-tier external validation, we report plainly that the signature is a strong classifier and a null prognosticator (Section 3.5 and Section 3.6). In a field where inflated single-cohort performance is common, this combination—exact interpretability, leakage-free validation, and transparent delineation of what a model can and cannot do—offers a transferable template for trustworthy explainable AI in precision oncology, consistent with emerging reporting standards [22].

### 4.5. Population Relevance for Saudi Colorectal Cancer

Although the models were trained and validated exclusively on international public cohorts, the convergence of the robustly enriched pathways with the Saudi CRC molecular landscape [21,58] provides a contextual bridge to a population that is under-represented in cancer genomics and that exhibits a distinct mutational profile, including a markedly lower *APC* and higher *TP53* mutation frequency than Western cohorts [21]. The convergence at the Wnt, PI3K, TP53 and RAS–MAPK pathways, and specifically the *CDH3*/Wnt link, indicates that the signature engages biology active in Saudi patients and identifies concrete, pre-specified targets for subsequent locally grounded validation. This contextualisation is deliberately framed as hypothesis-anchoring rather than validation, since no Saudi transcriptomic data were used at any stage.

### 4.6. Limitations

Several limitations bound these conclusions. First, the high classification accuracy, although externally replicated, reflects the large transcriptomic distance between tumour and adjacent-normal tissue and must not be read as clinical readiness; the harder tasks of separating tumour from pre-malignant lesions, or detecting disease from liquid biopsy, were beyond the present scope. Second, the panel is explicitly not prognostic, and the per-stage survival analyses were underpowered (EPV < 10) and reported only as exploratory. Third, four of the ten genes remain poorly characterised in CRC and are hypothesis-generating rather than validated. Fourth, the Saudi link is at the pathway level only, as no Saudi or Middle Eastern transcriptomic cohort was available for direct validation. Fifth, bulk transcriptomics cannot resolve the cellular source of signals such as *ESM1* (endocan); as an endothelial cell-specific molecule elevated in colorectal tumours [56], part of this signal plausibly derives from the tumour vasculature rather than the epithelial compartment. Finally, the cross-platform external stage validation (microarray validation of an RNA-seq-derived signature) reflects the absence of a suitably staged public RNA-seq cohort rather than a design choice and represents the strongest external stage-level test the available public data permit.

### 4.7. Future Directions

These limitations define a concrete programme of future work. The immediate priority is prospective validation in a Saudi CRC transcriptomic cohort, which would convert the present pathway-level population link into direct evidence. The reproducible late-stage *ESM1* signal motivates a focused serum-endocan study across AJCC stages, exploiting its secreted nature for a minimally invasive assay. Single-cell or spatial transcriptomics would resolve the cellular origin of the stage-dependent drivers and test the tumour-endothelium hypothesis for *ESM1*, while the four uncharacterised candidate genes warrant targeted experimental follow-up. More broadly, the stage-stratified explainability framework is disease-agnostic: applying it to other staged malignancies would test whether stage-dependent shifts in feature importance are a general property of progressing tumour transcriptomes, potentially establishing a new, interpretability-driven mode of biomarker discovery. We emphasise a conservative view of clinical applicability—future steps would involve prospective validation in other cohorts, assay standardisation using a pre-defined decision threshold, direct transcriptomic validation in the target population (including a Saudi CRC cohort), and evaluation in representative demographically diverse populations. The present work is specifically a retrospective computational validation to create a reproducible framework and is not a clinically deployable test.

## 5. Conclusions

This study advances colorectal-cancer transcriptomic modelling from static prediction toward a stage-resolved, interpretability-driven analysis. Three findings define its contribution. Conceptually, we demonstrate for the first time in CRC that the feature-importance structure of an explainable classifier is itself stage-dependent and externally replicable, with *ESM1* emerging as a reproducible, mechanistically supported late-stage driver surfaced by the movement of its attribution rather than by expression alone. Methodologically, the use of an exactly interpretable model, a leakage-free protocol, multi-cohort validation, and a pre-registered prognostic assessment allows the signature’s scope to be delineated transparently—a strong, generalisable tumour-versus-normal classifier that is deliberately not advanced as a prognostic model. Biologically, the signature proves complementary to classical network hubs, recovering genes that connectivity-based methods would overlook, and its enriched pathways converge with the Saudi colorectal-cancer landscape, anchoring future population-specific validation. By prioritising exact interpretability, reproducibility and transparent scope over inflated performance, the work offers a transferable template for trustworthy explainable artificial intelligence in precision oncology.

## Figures and Tables

**Table 1:** Critical evaluation of representative prior literature on colorectal cancer transcriptomic biomarkers, explainable AI methodology, stage-specific molecular progression, and Saudi population data. Studies assessed the topic area, key strengths, weaknesses and limitations, potential sources of bias, and the specific gap each leaves unaddressed. The rightmost column identifies which gap is addressed by the present study. Studies are ordered by methodological relevance to the present work.

Study/Year	Topic Area	Strengths	Weaknesses/ Limitations	Potential Bias	Gap Addressed by Present Study
Wu et al. 2025 [1]/Bray et al. 2024 [2]	Global CRC epidemiology (GLOBOCAN)	Comprehensive incidence/mortality estimates across 185 countries; projections to 2050	Aggregate registry data; no molecular or transcriptomic dimension; regional under-sampling in MENA	Reporting bias toward high-income-country registries with mature data infrastructure	No transcriptomic or population-specific biomarker dimension; motivates the present multi-cohort design
Alessa & Khan 2024 [4]	Saudi CRC epidemiology review	Synthesises 16 studies on Saudi-specific incidence, risk factors, and outcomes	Narrative review; no primary molecular data; no computational modelling	Selection limited to English-language, PubMed-indexed studies	Confirms population-specific epidemiological divergence motivating Contribution 2
Alhurishi et al. 2024 [6]	Saudi CRC registry cohort study	Large registry-based sample; identifies risk factors for late-stage presentation in Saudi patients	Clinical/demographic covariates only; no molecular or gene-expression data examined	Registry under-capture of rural/remote cases possible	Confirms late-stage presentation pattern motivating population-context discussion (Contribution 2)
Alsolme et al. 2023 [21]	Saudi CRC targeted NGS mutation panel (*n* = 107)	Large dedicated Saudi cohort; actionable-gene mutation frequencies reported (*BRCA2* 79%, *CHEK1* 78%, *ATM* 76%, *PMS2* 76%, *ATR* 74%, *MYCL* 73%)	Targeted DNA mutation panel only—no transcriptomic/expression data; no SHAP or explainability framework; cohort not linked to any public expression repository	Single-country, two-centre recruitment (KFMC, KAUH) may not capture full national diversity	No transcriptomic cohort from this population exists publicly; present study instead cross-references these mutation frequencies against SHAP-prioritised genes in Discussion (Contribution 2)
Phipps et al. 2020 [9]	CMS molecular subtyping, pooled analysis	Demonstrates transcriptome-derived subtypes carry prognostic value beyond TNM stage	Subtype labels are categorical; no per-gene explainability; no stage-resolved attribution	Pooled cohorts predominantly Western/European ancestry	No SHAP-based, stage-resolved transcriptomic attribution; addressed by Contribution 1
Yin et al. 2026 [13]	ML + SHAP CRC diagnostic model (TCGA + GSE87211)	Nine classifiers benchmarked; XGBoost AUC = 0.990; SHAP identifies *IFITM1, DBNDD1* as top contributors	Cohort-averaged SHAP only; no stage stratification; no survival modelling; no external cohort beyond validation split	Mendelian-randomisation gene set may not capture all clinically relevant biomarkers	No stage-resolved SHAP; no survival benchmarking—addressed by Contributions 1 and 4
Wei et al. 2023 [14]	ML + SHAP tumour micro-environment biomarkers (TCGA/GEO)	Four feature-selection algorithms benchmarked; SHAP-derived co-predictive network visualised via VisuNet; PPI network from STRINGdb	Single-database enrichment context; no stage-stratified SHAP; no external cohort beyond validation split	Cohort restricted to TCGA/GEO without population diversification	No stage-stratified attribution; single-source mechanistic linkage only—addressed by Contributions 1 and 3
Kollampallath Radhakrishnan et al. 2025 [15]	SHAP-based plasma proteomic biomarkers (UK Biobank)	Large biobank sample; three classifiers compared; SHAP identifies *TFF3*, *LCN2*, *CEACAM5* with pathway linkage	Proteomic, not transcriptomic, layer; UK Biobank population only; no stage stratification	UK Biobank cohort skews toward healthier, more affluent volunteers	No transcriptomic SHAP equivalent with population diversity beyond the UK; no stage-level analysis
Poorhasani & Iancu 2026 [16]	Stage-specific SHAP/LIME survival prediction (CRC, gastric, liver)	First demonstration that top SHAP-ranked predictors shift materially across localised, regional, and distant stage	Entirely clinical/demographic covariates (age, nodal counts, treatment codes); no transcriptomic features examined	SEER registry coverage biased toward US healthcare-access patterns	Stage-dependent SHAP shift not yet demonstrated at the gene-expression level—directly addressed by Contribution 1
Alhosani et al. 2025 [17]	absGSEA stage-specific transcriptomics (adenoma → CIS → adenocarcinoma)	Whole-transcriptome RNA-seq on FFPE tissue across three progression stages; RT-qPCR experimental validation	Gene-set enrichment approach, not predictive ML; no SHAP or feature-level attribution; no survival benchmarking	Small per-stage sample sizes (*n* = 8–11 per group)	Confirms stage-specific transcriptomic signal exists, but no SHAP-based explainable model has been built on it—addressed by Contribution 1
Palaniappan et al. 2016 [18]	Stage-specific driver-gene network analysis (CRC)	Identifies distinct hub driver genes for Stage II, III, and IV progression via network centrality	Network-centrality approach only; no explainable-AI attribution; no model-based prediction	Driver-gene calls dependent on chosen network-construction threshold	No SHAP-based explainability layer applied to stage-specific gene sets—addressed by Contribution 1
Shabani et al. 2022 [19]	qRT-PCR validation of stage-dependent gene expression (CRC)	Direct wet-lab confirmation that FOXM1, hTERT, MCTP1 show stage-dependent expression across Stage I–IV	Small cohort (*n* = 54); single-centre; targeted gene panel, not genome-wide; no ML model	Single-institution sample may not generalise across populations	Confirms biological plausibility of stage-dependent transcription, supporting the rationale for Contribution 1
Collins et al. 2024 [22]	TRIPOD + AI reporting guideline	Defines minimum reporting standard for clinical prediction models using ML, including external validation and calibration	Guideline only; does not itself supply biomarkers or models; compliance burden may be high for exploratory studies	Guideline development panel weighted toward high-resource health systems	Existing CRC SHAP literature inconsistently meets these standards—addressed by Contribution 4
Lundberg & Lee 2017 [23]	SHAP unified framework	Exact Shapley-value attributions; satisfies efficiency, symmetry, and dummy axioms; model-agnostic	Computationally expensive for large ensembles; attribution stability across random seeds not addressed by the original framework	Model-specific SHAP variants can yield different rankings for the same data	First application of stage-stratified SHAP attribution within a single CRC transcriptomic model—addressed by Contribution 1

**Table 2:** Justification for the method family selected at each stage of the pipeline.

Pipeline Component	Method Family Used	Justification
Primary classification (tumour vs. normal; per-stage modelling)	Benchmarked comparison across six algorithms; final choice empirically justified, not assumed	Rather than selecting XGBoost a priori, six candidate algorithms spanning linear, kernel, bagged-tree, boosted-tree, and shallow neural network families are trained under identical cross-validation folds (Section 2.5.1) and compared on external validation AUC with DeLong’s test; the winning model is then used for all downstream SHAP attribution. This directly addresses the reasonable concern that a single default algorithm should not be assumed superior without comparison.
Explainability layer	SHAP (TreeExplainer [25] for tree-based winners; KernelExplainer if a non-tree model wins)	TreeExplainer provides exact Shapley values for tree ensembles in polynomial time; if a non-tree model (e.g., Elastic Net or the shallow MLP) is selected instead, the corresponding exact or near-exact explainer (LinearExplainer or DeepExplainer) is substituted, preserving theoretical fidelity regardless of which model wins the benchmark.
Mechanistic/interaction layer	Graph-based network analysis (STRINGdb [30] + MCODE [31])—NOT a graph neural network	The objective here is descriptive network topology (hub identification, module detection) on a few hundred prioritised genes, a setting where established graph-theoretic clustering algorithms (MCODE) are standard, interpretable, and require no training. A graph neural network was explicitly considered and not pursued: at *n* ≈ 350–450 there is insufficient data to train a GNN that would be expected to outperform deterministic network analysis, and including an underpowered GNN comparator purely for completeness would itself be methodologically misleading. This decision is stated explicitly here and revisited as a stated limitation in the Discussion, rather than left unaddressed.
Survival modelling	Cox proportional hazards (baseline) benchmarked against DeepSurv [43] and Random Survival Forest [44]	Deep learning is intentionally introduced here, but only as one of three benchmarked candidates, not as a default choice. Per-stage event counts are checked against the established events-per-variable (EPV) heuristic [42] before any stage-specific survival model is reported with full confidence; stage subgroups falling below this threshold are flagged as exploratory rather than confirmatory.

**Table 3:** Six-algorithm benchmark: model families compared and their role in the comparison.

Model	Family	Role in Benchmark	Hyperparameters Tuned (Optuna, Same Budget per Model)
Logistic Regression (Elastic Net)	Linear ML [23]	Interpretability floor; tests whether non-linearity is needed at all	L1 ratio, regularisation strength C
Random Forest	Bagged trees [36]	Tests whether boosting specifically (vs. ensembling generally) adds value	n_estimators, max_depth, min_samples_leaf
Support Vector Machine (RBF kernel)	Kernel ML [38]	Common comparator in the existing CRC-SHAP literature; non-linear, non-tree alternative	C, gamma
XGBoost	Boosted trees [13]	Current default in the literature; primary candidate	Per Section 2.5.1
LightGBM	Boosted trees [37]	Faster alternative boosting implementation; tests sensitivity to boosting library choice	num_leaves, learning_rate, n_estimators
Shallow MLP (1–2 hidden layers)	Deep learning, deliberately small [39]	Directly tests ‘why not deep learning’ with evidence; deliberately not over-parameterised given *n* ≈ 350–450	hidden_layer_sizes, dropout, learning_rate, L2 weight decay

**Table 4:** Hyperparameter search space and tuning protocol (shown for XGBoost; equivalent search spaces are defined for each of the other five benchmarked models).

Hyperparameter	Search Range/Method	Notes
n_estimators	100–1000 (Optuna [40], log-uniform)	Early stopping on inner-fold validation AUC, patience = 30 rounds
max_depth	3–10 (integer)	Shallow trees favoured given HDLSS regime; penalises deep, overfit splits
learning_rate (eta)	0.001–0.3 (log-uniform)	Jointly tuned with n_estimators via Optuna Bayesian search (100 trials)
subsample	0.5–1.0	Row subsampling per boosting round to reduce variance
colsample_bytree	0.3–1.0	Column subsampling; important given the wide feature-to-sample ratio
min_child_weight	1–10	Minimum sum of instance weight needed in a child; controls overfitting
gamma	0–5.0	Minimum loss reduction for further partition; additional regularisation
reg_alpha/reg_lambda	0–10.0 (L1/L2)	Elastic-net-style regularisation on leaf weights
random_state	Fixed at 42 throughout	Applied to data splits, all six models’ training, SHAP background sampling, and bootstrap resampling for full reproducibility

**Table 5:** Methodological risks identified prior to data acquisition and their adopted resolutions.

#	Issue	Resolution Adopted in This Study
1	Cross-platform mixing (RNA-seq vs. microarray) can shrink common gene overlap below 100 genes after harmonisation [29]	Primary analysis restricted to RNA-seq cohorts only (TCGA-COAD plus RNA-seq GEO series). Microarray-based cohorts (e.g., GSE39582, GSE17536 [49], used as external validation) are kept as a separate, explicitly labelled validation layer rather than merged into the training matrix, avoiding the common-feature collapse documented in the platform-mixing literature.
2	Stage-stratified subgroups, particularly Stage I (*n* = 45), may be too small for stable SHAP attribution	Three layered safeguards: (i) no model is trained on any single small subgroup—SHAP attributions are a post hoc stratification of one full-cohort model (Section 2.5.4); (ii) a minimum-n-per-stage threshold (*n* ≥ 30) and a 1000-iteration bootstrap SHAP-ranking stability check (top-10 Spearman ρ > 0.7) are pre-specified; (iii) a pre-registered fallback collapses the analysis to well-powered early-stage (I–II) versus late-stage (III–IV) groups if any subgroup fails the stability bar, with the outcome of this rule reported explicitly either way (Section 2.5.4).
3	Low event (death) counts per stage may violate the events-per-variable heuristic for survival modelling [42]	Actual event counts per AJCC stage are tabulated immediately after data acquisition (Section 2.2.1) and reported transparently. Stage-specific survival benchmarking is reported as confirmatory only for stages meeting the conventional EPV ≥ 10 threshold; stages below this are explicitly labelled exploratory.
4	Multiple-testing burden across four stage-stratified DEG analyses could inflate false-positive ‘stage-specific’ findings	Benjamini–Hochberg FDR correction [41] is applied within each stage-specific analysis and is also reported after a second, more conservative correction pooling all stage-by-gene tests performed across the study, so that the reader can see results under both the per-stage and the pooled multiple-testing regime.
5	Saudi pathway cross-reference (Section 2.6) could be perceived as selectively reported only if favourable	This Methods section pre-commits, in writing, to reporting the outcome of the Saudi pathway comparison regardless of direction—convergence or non-convergence are both reported in the Results and Discussion, and this pre-commitment is stated here prior to any analysis being run.
6	ICGC data portal was retired in June 2024; ICGC controlled-access data (including detailed clinical/survival annotation) now requires DACO approval and is not freely downloadable [47]	ICGC COAD-READ is replaced as the external validation cohort. External validation is instead performed on GSE39582 and GSE17536/GSE17537 [49], established, fully open-access GEO cohorts with complete survival annotation that have been used as external validation in multiple published CRC prognostic studies, requiring no data access committee approval.
7	AJCC staging edition may differ across cohorts and collection years, risking silent stage mismatches when merging	The AJCC edition associated with each cohort’s clinical metadata [8] is recorded explicitly during data acquisition (Section 2.2.1); where a cohort’s documentation does not specify edition, conservative re-mapping to AJCC 8th edition stage groups (collapsing sub-stages, e.g., IIIA/IIIB/IIIC → III) is applied uniformly before any cross-cohort stage-stratified analysis.
8	Public GitHub/code-release plans require co-author review and a settled licence before submission	MIT licence is adopted for all released code (consistent with the existing PyImpetus-SHAP repository precedent); the repository is shared with co-author Sultan Alhayyani for review prior to being made public, and this step is scheduled before, not during, manuscript submission.

**Table 6:** Audited inventory of study limitations, each tested for whether it is fixable within scope. Only genuinely unavoidable constraints, or inherent residuals remaining after the doable portion has been performed, are carried to the manuscript’s Limitations section (Section 4.6).

Limitation	Doability Assessment	Action Taken/Status
External validation of the stage-stratified attribution	PARTLY DOABLE—GSE39582 carries full TNM staging, so a cross-platform stage-stratified external validation is achievable; only same-platform (RNA-seq) stage validation is impossible because no such public cohort exists.	Doable part performed: stage-stratified signature transferred to and validated on GSE39582 (Section 2.2.2, Tier 2). Residual declared: external stage validation is cross-platform, not same-platform.
Absence of prospective wet-lab validation of prioritised genes	PARTLY DOABLE—prospective qRT-PCR/IHC is outside computational scope, but two computational corroboration steps are doable: cross-referencing against published experimental evidence, and confirming expression direction in an independent transcriptomic cohort.	Doable parts performed: literature-based corroboration (Section 2.6.1) and independent-cohort expression-direction confirmation (Section 2.6.2). Residual declared honestly: both remain within or borrow from existing evidence and are not orthogonal wet-lab validation of this study’s specific findings; prospective experimental validation flagged as future work.
No Saudi transcriptomic cohort for training or validation	TRULY UNAVOIDABLE—no public Saudi CRC transcriptomic dataset exists; generating one requires wet-lab work outside scope.	Doable part performed: pathway-level contextualisation against published Saudi mutation data (Section 2.6). Residual declared as a genuine, unavoidable limitation.
Single primary training cohort (TCGA-COAD only)	EFFECTIVELY UNAVOIDABLE—candidate RNA-seq augmentation cohorts identified were too small or lacked stage labels; forcing them in would introduce batch artefacts without meaningful gain.	Declared explicitly with justification that available RNA-seq cohorts were inadequate for augmentation; mitigated by rigorous internal nested CV and multi-tier external validation.
Small Stage I subgroup (*n* = 45)	PARTLY DOABLE—the doable safeguards (no per-subgroup training, bootstrap stability check, and early-vs-late fallback) are implemented; residual small-n is inherent.	Doable parts performed (Section 2.5.3 and Section 2.5.4). Residual inherent small-n declared.
Cross-sectional design; no longitudinal expression trajectories	TRULY UNAVOIDABLE—TCGA and the validation cohorts are cross-sectional by construction.	Declared as a genuine limitation; longitudinal validation noted as future work.
SHAP attribution reflects association, not proven biological causation	TRULY UNAVOIDABLE—a property of attribution methods; no computational step converts attribution into causal proof.	Declared honestly to avoid mechanistic overclaim; causal interpretation explicitly disclaimed.

**Table 7:** Imbalance-robust performance metrics for the benchmarked classifiers, with weighted and unweighted variants of the selected model. Near-maximal AUPRC and the negligible effect of class weighting provide evidence that the ≈7:1 imbalance did not bias classification. Full per-model values and bootstrap CIs are given in Figure 4 and the released code.

Model (Variant)	AUC	AUPRC	MCC	Balanced Accuracy
Elastic Net (unweighted)	1.000	1.000	1.000	1.000
Elastic Net (weighted)	1.000	1.000	0.988	0.998
Random Forest (unweighted)	1.000	1.000	1.000	1.000
LightGBM (unweighted)	1.000	1.000	1.000	1.000
XGBoost (unweighted)	0.987	0.997	0.985	0.988
MLP (unweighted)	0.987	0.998	0.756	0.942

## Data Availability

All data analysed in this study are publicly available: TCGA-COAD via the UCSC Xena platform; GSE50760, GSE39582 and GSE17536 via the Gene Expression Omnibus. All analysis code, processed data, trained models, and figure-generation scripts are released under the MIT licence in the project repository (https://github.com/SAH-ML/crc-stage-stratified-xai, accessed on 11 October 2026), enabling complete reproduction of every result.

## Supplementary Materials

The following supporting information can be downloaded at: https://www.mdpi.com/article/10.3390/biomedicines14102375/s1. Main-text figures are Figure 1, Figure 2, Figure 3, Figure 4, Figure 5, Figure 6, Figure 7, Figure 8 and Figure 9. The items below are confirmatory, sensitivity, or large tabular content supporting but not carrying the principal narrative. Table S1: Differential-expression concordance between the canonical R-limma and Python implementations (global and per-stage). Table S2: Differential-expression statistics for the ten panel genes in both implementations. Table S3: Full triangulated enrichment table (817 pathways) with per-platform significance and Benjamini–Hochberg FDR corrections (provided as a separate machine-readable CSV file). Table S4: Two-level network analysis of the panel genes (degree, betweenness and neighbours at STRING confidence > 0.9 and > 0.7). Table S5: Panel-gene membership in the robustly enriched pathways. Table S6: Saudi colorectal-cancer pathway cross-reference with literature sources. Table S7: Literature corroboration of the ten panel genes (status, direction, references). Table S8: Stage-wise bootstrap 95% confidence intervals for the SHAP concordance statistics. Table S9: Cohort characteristics and per-cohort (non-pooled) performance. Table S10: Permutation test and repeated nested cross-validation of the classifier. Table S11: Robustness of the *ESM1* stage association to adjustment for the other panel genes. Figure S1: Per-stage exploratory survival analyses (overall-survival discrimination across models; all subgroups EPV < 10). Figure S2: Network-confidence sensitivity analysis (STRING confidence >0.9 vs. >0.7). Figure S3: GEPIA2 expression box plots for the ten panel genes (tumour vs. normal, TCGA + GTEx reference). Figure S4: Permutation test of the classifier: observed AUC versus the permuted-label null distribution.

## Abbreviations

Abbreviation	Definition
AJCC	American Joint Committee on Cancer
AUC	Area under the receiver–operating–characteristic curve
AUPRC	Area under the precision–recall curve
CI	Confidence interval
C-index	Concordance index
COAD	Colon adenocarcinoma
CRC	Colorectal cancer
DEG	Differentially expressed gene
EPV	Events per variable
FDR	False discovery rate
GTEx	Genotype-Tissue Expression project
log2FC	Log2 fold-change
MCC	Matthews correlation coefficient
MCODE	Molecular Complex Detection
OS	Overall survival
PPI	Protein–protein interaction
RSF	Random survival forest
SHAP	SHapley Additive exPlanations
TCGA	The Cancer Genome Atlas
TNM	Tumour–Node–Metastasis
XAI	Explainable artificial intelligence
