# Supplementary material for: Stage-Stratified Explainable Machine Learning Identifies a Reproducible Transcriptomic Classifier with Stage-Dependent Feature Importance in Colorectal Cancer: A Multi-Cohort Study with Saudi Population Contextualisation
Source: Biomedicines. 2026 Oct 18;14(10):2375. doi: 10.3390/biomedicines14102375 (PMC13701872; doi:10.3390/biomedicines14102375)
Supplement: Supplementary file 1 [file biomedicines-14-02375-s001.zip › Paper1_Supplementary_REVISED_Biomedicines.pdf]

Supplementary Materials

Stage-Stratified Explainable Machine Learning Identifies a Reproducible Transcriptomic Classifier with Stage-Dependent Feature Importance in Colorectal Cancer: A Multi-Cohort Study with Saudi Population Contextualisation

Table S1. Differential-expression concordance between the canonical R-limma and Python implementations (global and per-stage).

Contrast	R DEGs	Python DEGs	Jaccard	log2FC Spearman	Direction agreement
Global	4,810	4,807	0.995	1.000	100%
Stage I	4,979	4,996	0.963	0.999	100%
Stage II	4,865	4,857	0.989	1.000	100%
Stage III	4,682	4,661	0.986	1.000	100%
Stage IV	4,971	4,971	1.000	1.000	100%

DEG counts at FDR < 0.05 and |log2FC| ≥ 1. The two independent implementations agree near-perfectly, establishing the differential-expression layer as implementation-independent.

Table S2. Differential-expression statistics for the ten panel genes in both implementations.

Gene	R log2FC	Python log2FC	R FDR	Direction match
CDH3	+6.548	+6.544	8.4×10^{-128}	Yes
OTOP2	−9.480	−9.475	8.4×10^{-128}	Yes
KRT80	+6.710	+6.713	2.6×10^{-109}	Yes
ETV4	+5.475	+5.476	1.3×10^{-101}	Yes
ESM1	+5.564	+5.563	2.2×10^{-99}	Yes
DHRS7C	−2.757	−2.756	1.2×10^{-115}	Yes
AADACL2	−2.761	−2.760	5.3×10^{-114}	Yes
LGI1	−4.567	−4.563	6.7×10^{-90}	Yes
TMEFF2	−3.344	−3.354	2.2×10^{-82}	Yes
KHDRBS2	−2.474	−2.472	1.2×10^{-77}	Yes

All ten panel genes are significant in the canonical limma analysis with concordant direction and near-identical fold-changes across implementations.

Table S4. Two-level network analysis of the panel genes (degree, betweenness and neighbours at STRING confidence > 0.9 and > 0.7).

Gene	Present at conf > 0.9	Present at conf > 0.7	Degree (0.7)
CDH3	No	Yes	2
OTOP2	No	Yes	1
LGI1	Yes	Yes	7
KRT80	No	No	0
ETV4	No	No	0
ESM1	No	No	0
DHRS7C	No	No	0

Gene	Present at conf > 0.9	Present at conf > 0.7	Degree (0.7)
AADACL2	No	No	0
TMEFF2	No	No	0
KHDRBS2	No	No	0

Panel genes are largely absent from or peripheral to the high-confidence STRING PPI network at both thresholds, and never interconnect, indicating complementarity to classical hub-gene approaches.

Table S5. Panel-gene membership in the robustly enriched pathways.

Gene	# robust pathways	Representative pathway(s)
CDH3	9	Wnt signalling; cell adhesion
LGI1	9	synaptic / axon-guidance (neural)
ESM1	5	angiogenesis; receptor signalling
DHRS7C	5	ion / metabolic homeostasis
ETV4	2	signal transduction; differentiation
OTOP2	1	ion transport
KRT80	1	epithelial structure
KHDRBS2	1	RNA binding
AADACL2	0	(none)
TMEFF2	0	(none)

Eight of ten panel genes belong to robustly enriched pathways; ESM1 maps to angiogenesis and CDH3 to Wnt/adhesion, linking the model's key genes to established CRC biology.

Table S6. Saudi colorectal-cancer pathway cross-reference with literature sources.

Saudi-altered pathway	Convergent	Saudi frequency / source
Wnt/β-catenin	Yes	65%; Alatwi et al. 2025 [58]
PI3K	Yes	48%; Alatwi et al. 2025 [58]
TP53	Yes	~50%; Alsolme et al. 2023 [21]
KRAS/RAS–MAPK	Yes	variable; [21,58]
Homologous recombination	No	61%; [58]

Four of five frequently altered Saudi CRC pathways converge with the robustly enriched pathways; the single non-convergence (homologous recombination) is reported transparently.

Table S7. Literature corroboration of the ten panel genes (status, direction, references).

Gene	Direction	Status	Representative evidence
KRT80	Up	Strong	Most up-regulated gene in TCGA CRC; metastasis [52]
OTOP2	Down	Strong	Down-regulated tumour suppressor in COAD [53]
CDH3	Up	Strong	P-cadherin overexpressed; poor prognosis [54]
ESM1	Up	Strong	Angiogenesis; advanced stage [55,56]
ETV4	Up	Strong	Pro-metastatic ETS factor [57]
TMEFF2	Down	Moderate	Methylation-silenced tumour suppressor
AADACL2	Down	Novel	Poorly characterised in CRC
KHDRBS2	Down	Novel	Poorly characterised in CRC

Gene	Direction	Status	Representative evidence
LGI1	Down	Novel	Limited CRC-specific evidence
DHRS7C	Down	Novel	Poorly characterised in CRC

Six of ten genes have direct experimental support in CRC, all matching the differential-expression direction; four are presented as hypothesis-generating candidates.

Note: *Table S3 (the full 817-pathway triangulated enrichment table) is provided as a separate machine-readable file (triangulated_enrichment_all.csv) owing to its length.*

Table S8. Stage-wise bootstrap 95% confidence intervals for the SHAP concordance statistics.

Comparison	Spearman rho	95% CI	p-value
Global vs Stage I	0.903	[0.610, 1.000]	0.001
Global vs Stage II	0.867	[0.437, 1.000]	0.002
Global vs Stage III	0.976	[0.811, 1.000]	0.001
Global vs Stage IV	0.879	[0.469, 1.000]	0.003
Stage I vs Stage II	0.842	[0.414, 1.000]	0.006
Stage I vs Stage III	0.83	[0.427, 0.987]	0.005
Stage I vs Stage IV	0.661	[0.074, 0.923]	0.038
Stage II vs Stage III	0.818	[0.412, 0.962]	0.008
Stage II vs Stage IV	0.636	[-0.057, 0.897]	0.047
Stage III vs Stage IV	0.927	[0.617, 1.000]	0.001

Point estimates are the Spearman correlations reported in the main text; 95% confidence intervals are from 1,000 bootstrap resamples (seed 42). The monotonic gradient is preserved: the extreme stages (Stage I vs IV) and Stage II vs IV show the weakest, widest-interval concordance, whereas adjacent late stages (Stage III vs IV) and all stage-vs-global comparisons are strong and tightly bounded, confirming that the stage-specific rankings are robust rather than driven by a few influential samples.

Table S9. Cohort characteristics and per-cohort (non-pooled) performance.

Cohort	Role	Platform	Samples	Staging	Performance
TCGA-COAD	Discovery / training	RNA-seq (Illumina HiSeq)	286 tumour / 41 normal	AJCC I–IV (45/110/80/39; 12 unstaged)	Internal AUC 1.000 (permutation p<0.001)
GSE50760	Tier 1: same-platform classification replication	RNA-seq	18 tumour / 18 normal	Primary + metastatic	External AUC 0.969 [0.896–1.000]; AUPRC 0.980
GSE39582	Tier 2: cross-platform stage + survival validation	Microarray (Affymetrix)	579 (full TNM staging)	AJCC I–IV (38/271/210/60)	Stage-importance concordance significant at 3/4 stages (rho up to 0.94); survival C-index 0.520 [0.477–0.565]
GSE17536	Tier 2: cross-platform survival validation	Microarray (Affymetrix)	177	With survival annotation	Survival C-index 0.575 [0.503–0.642]

Each external cohort is reported separately, reflecting its distinct platform, size, clinical composition, and validation role. GSE50760 is a small cohort (18 tumour, 18 normal), so its external AUC estimate carries a correspondingly wide confidence interval and is interpreted as supportive rather than definitive.

Table S10. Permutation test and repeated nested cross-validation of the classifier.

Analysis	Result
Observed 5-fold CV AUC	1.000
Permutation null AUC (1,000 label shuffles): mean	0.501

Permutation null AUC: 95th percentile	0.601
Permutation null AUC: maximum	0.690
Permutation p-value	0.000999
Repeated nested CV (10 seeds): mean AUC	1.000
Repeated nested CV: SD / range	0.000 / 1.000–1.000

The observed classification AUC lies far beyond the permuted-label null distribution (permutation $p < 0.001$; null mean 0.50, null maximum 0.69), and is invariant across ten independent cross-validation seeds, indicating that the near-perfect internal discrimination reflects a genuine, stable tumour-versus-normal signal rather than sample-specific structure or optimistic bias. See Figure S4.

Table S11. Robustness of the ESM1 stage association to adjustment for the other panel genes.

Test	Statistic	p-value
ESM1 expression vs AJCC stage (unadjusted)	Spearman rho = 0.123	0.040
ESM1 vs stage, partial (controlling other 9 panel genes)	partial rho = 0.120	0.045
ESM1 late (III+IV) > early (I+II) expression	median 7.09 to 7.25	0.062

The association between ESM1 expression and advancing AJCC stage is modest but retains significance after partial-correlation adjustment for the other nine panel genes (partial rho = 0.120, p = 0.045), indicating an independent stage-associated component rather than mere co-variation with the broader advanced-stage programme. Consistent with the cautious interpretation adopted in the main text, the effect size is small; full adjustment for tumour-microenvironment composition would require cell-type deconvolution data not available for this bulk cohort and is identified as a next step.

Supplementary Figures

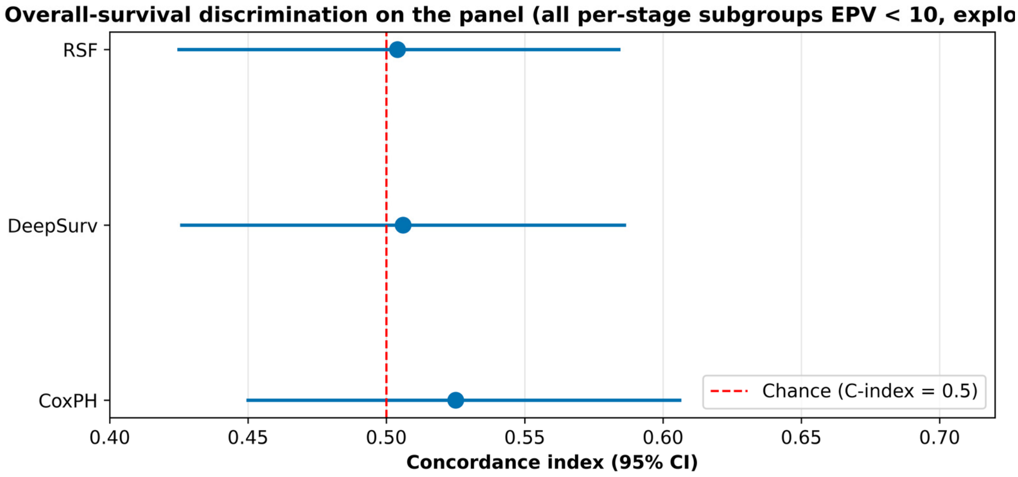

Figure S1. Per-stage exploratory survival analyses (overall-survival discrimination across models; all subgroups EPV < 10).

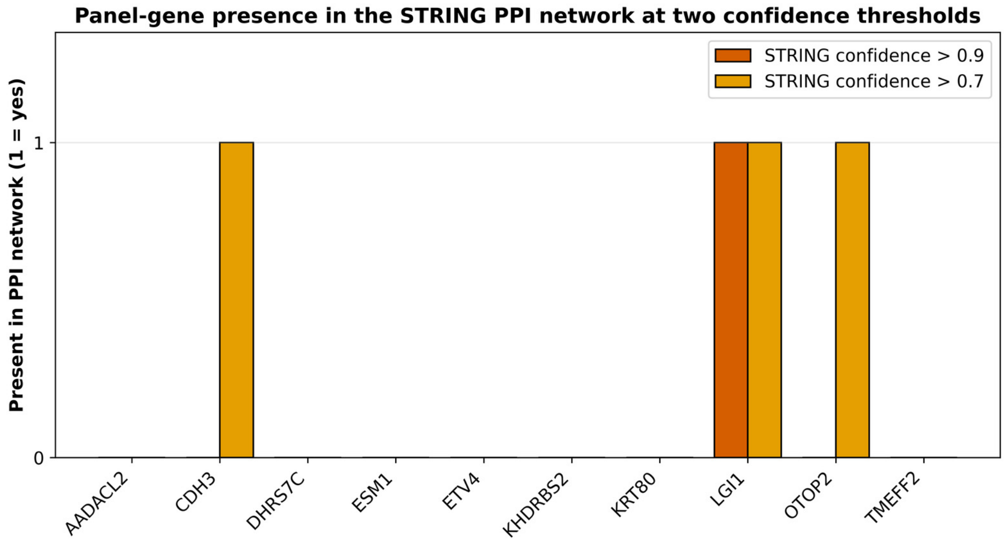

Figure S2. Network-confidence sensitivity analysis (STRING confidence > 0.9 vs. > 0.7).

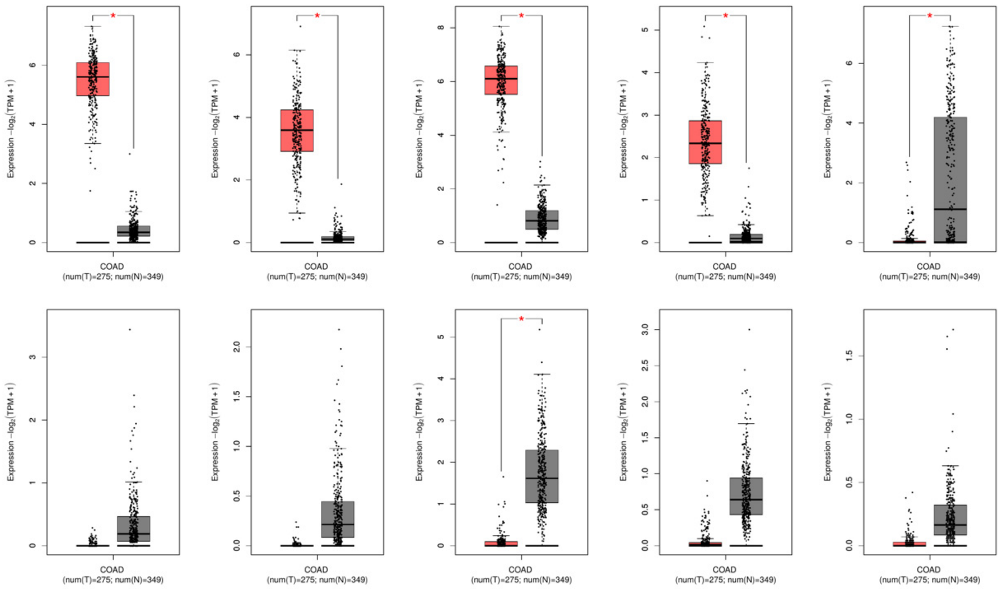

Figure S3. GEPIA2 expression box plots for the ten panel genes (tumour vs. normal, TCGA + GTEx reference).

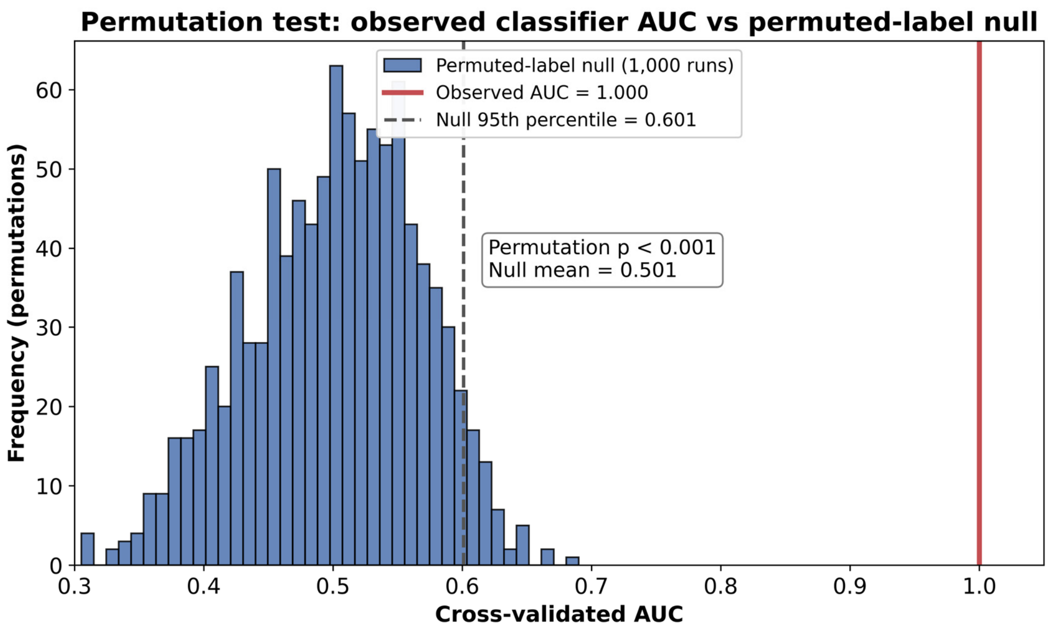

Figure S4. Permutation test of the classifier: observed AUC versus the permuted-label null distribution.

Observed cross-validated AUC (red line) versus the null distribution obtained by shuffling the class labels 1,000 times and re-running the full leakage-free pipeline. The observed value lies far above the null 95th percentile (0.60) and null maximum (0.69), with permutation p < 0.001.
